# diceCT: A Valuable Technique to Study the Nervous System of Fish

**DOI:** 10.1523/ENEURO.0076-20.2020

**Published:** 2020-08-13

**Authors:** Victoria Camilieri-Asch, Jeremy A. Shaw, Andrew Mehnert, Kara E. Yopak, Julian C. Partridge, Shaun P. Collin

**Affiliations:** 1 School of Biological Sciences, The University of Western Australia, Perth, 6009 Western Australia, Australia; 2 Oceans Institute, The University of Western Australia, Perth, 6009 Western Australia, Australia; 3 Centre for Microscopy, Characterisation and Analysis (CMCA), The University of Western Australia, Perth, 6009 Western Australia, Australia; 4 Department of Biology and Marine Biology and the Center for Marine Science, University of North Carolina Wilmington, Wilmington, NC 28409; 5 School of Life Sciences, La Trobe University, Melbourne, 3086 Victoria, Australia

**Keywords:** diceCT, comparative neuroanatomy, elasmobranch, teleost, iodine, X-ray tomography

## Abstract

Contrast-enhanced X-ray imaging provides a non-destructive and flexible approach to optimizing contrast in soft tissues, especially when incorporated with Lugol’s solution (aqueous I_2_KI), a technique currently referred to as diffusible iodine-based contrast-enhanced computed tomography (diceCT). This stain exhibits high rates of penetration and results in excellent contrast between and within soft tissues, including the central nervous system. Here, we present a staining method for optimizing contrast in the brain of a cartilaginous fish, the brownbanded bamboo shark, *Chiloscyllium punctatum*, and a bony fish, the common goldfish, *Carassius auratus*, using diceCT. The aim of this optimization procedure is to provide suitable contrast between neural tissue and background tissue(s) of the head, thereby facilitating digital segmentation and volumetric analysis of the central nervous system. Both species were scanned before staining and were rescanned at time (T) intervals, either every 48 h (*C. punctatum*) or every 24 h (*C. auratus*), to assess stain penetration and contrast enhancement. To compare stain intensities, raw X-ray CT data were reconstructed using air and water calibration phantoms that were scanned under identical conditions to the samples. Optimal contrast across the brain was achieved at T = 240 h for *C. punctatum* and T = 96 h for *C. auratus*. Higher resolution scans of the whole brain were obtained at the two optimized staining times for all the corresponding specimens. The use of diceCT provides a new and valuable tool for visualizing differences in the anatomic organization of both the central and peripheral nervous systems of fish.

## Significance Statement

This study uses an emerging bioimaging technique based on X-ray CT, to advance our visualization, and thus understanding, of brain organization and structure in a cartilaginous and bony fish model species. Diffusible iodine-based contrast-enhanced computed tomography (diceCT) is a relatively new approach to improving contrast between/within soft tissues and is becoming increasingly popular across many fields of research, including evolutionary biology, biomechanics, paleontology, and comparative anatomy. Few studies have undertaken a rigorous assessment of stain penetration using Lugol’s iodine, especially in the brain. Here, we present the first staining optimization using diceCT in any species of fish. There is therefore a vital need to standardize the methodology used, in order to collect comparable, replicable data, and thus further develop its usage and value across disciplines and taxa.

## Introduction

Imaging the central nervous system (or brain) of animals can reveal vital information about the organization and the relative size of sensory brain regions (Buschhüter et al., 2008; Yopak, 2012). The volume of major brain subdivisions—olfactory bulbs, telencephalon, diencephalon, mesencephalon, cerebellum, and medulla oblongata—relative to the total volume of the brain, has been used to assess evolutionary adaptations and ontogenetic shifts in sensory capabilities across a wide range of vertebrate taxa, including fish (Brandstätter and Kotrschal, 1990; Wagner, 2003; Lisney and Collin, 2006; Lisney et al., 2007; Yopak et al., 2007; Yopak and Frank, 2009; Eifert et al., 2015; Kotrschal et al., 2017; Axelrod et al., 2018). However, there are limited quantitative, high-resolution data on the organization of brain subdivisions in fish compared with other vertebrates, with cartilaginous fish receiving even less attention than other fish taxa, despite their basal position in the phylogenetic history of vertebrates (Yopak et al., 2007; Yopak, 2012; Yopak and Lisney, 2012).

To visualize and quantitatively measure soft tissues such as the brain, magnetic resonance imaging (MRI) and X-ray micro-computed tomography (μCT) are the two main techniques used for non-invasive imaging *in situ*. Historically, MRI has been preferred for soft tissue imaging, and it is currently the standard imaging technique in the field of fish neuroecology (Yopak and Frank, 2009; Ullmann et al., 2010b; Yopak et al., 2018), as it enables scanning of larger specimens (i.e., when the field of view is a limiting factor). Additionally, contrast agents commonly used in MRI (e.g. ProHance ^⋀^; Yopak and Frank, 2009) have not been reported to cause significant tissue shrinkage for the study of brain volumes, as opposed to staining agents required to increase contrast for μCT. However, the accessibility, cost, relative complexity and the duration of data acquisition for MRI can be limiting (Ullmann et al., 2010b), particularly for studies that require higher numbers of replicates, or scanning at higher resolutions, e.g. for small preserved specimens (when the field of view is not a limiting factor). Therefore, μCT is an attractive alternative, which offers the potential to resolve and quantitatively examine the organization of the brain and its major subdivisions, in a broad range of animals not suited to MRI.

X-ray imaging is typically useful for visualizing highly X-ray attenuating structures, such as bone, dentin and enamel, mineralized cartilage, chitin. However, to visualize weakly X-ray attenuating soft tissues, such as the brain, some form of contrast enhancement is required. Heavy metal contrast agents, such as osmium tetroxide (OsO_4_), phosphomolybdic acid (PMA), phosphotungstic acid (PTA), and iodine (I_2_), can be used to modify/enhance X-ray attenuation (Metscher, 2009a,b; Mizutani and Suzuki, 2012; Pauwels et al., 2013; Descamps et al., 2014). While the mechanisms by which these stains bind to tissues is are not fully understood, much of their utility is based on their rates of penetration, contrast enhancement, cost, and toxicity (Pauwels et al., 2013; Descamps et al., 2014; for review, see Mizutani and Suzuki, 2012; Gignac and Kley, 2014). In comparison with alternatives, iodine is convenient to administer, less toxic than osmium, and cost-effective. Importantly, iodine effectively highlights the overall structure and soft tissues of small samples (<1 cm^3^). However, in its simple form, elementary I_2_ can be less effective at staining deep soft tissues, such as the brain, especially in larger samples (>1 cm^3^), in a practical timeframe (unpublished data). A more effective iodine-based contrast agent is thus required to increase penetration rate and contrast in soft tissues.

Diffusible iodine-based contrast-enhanced CT (diceCT; Gignac et al., 2016) is a technique that combines μCT and the use of an iodine-based staining solution as the contrast agent, such as Lugol’s iodine (Metscher, 2009a,b; Degenhardt et al., 2010; Jeffery et al., 2011; Gignac and Kley, 2014). Lugol’s iodine (or I_2_KI-dH_2_O) is an aqueous solution of one unit weight per volume (w/v) of I_2_ with two units w/v of potassium iodide (KI) in distilled water (dH_2_O), which exhibits high rates of penetration and has been shown to greatly enhance the contrast between and within different types of soft tissue (Metscher, 2009a,b; Gignac et al., 2016). Other iodine-based solutions have been used successfully for contrast-enhanced μCT imaging, including I_2_-ethanol (I_2_E), I_2_-methanol (I_2_M), and KI, although their performance have not been directly compared with the one of Lugol’s iodine, to our knowledge. Lugol’s iodine appears to be the best performing staining solution for brain tissues of larger, postembryonic specimens (>1–2 cm), as it shows good tissue penetration and differentiation throughout samples (including deeper brain areas), despite the shrinkage observed (although less shrinkage seems to occur in denser brain regions, such as the cerebellum; Buytaert et al., 2014). Lugol’s iodine is of specific interest in neurobiological research, as it enables one to differentiate myelinated and unmyelinated neuronal tissue within both the central and peripheral nervous systems in vertebrates (Efimova et al., 2013; Gignac and Kley, 2014, 2018). μCT imaging using Lugol’s solution has been increasingly used in recent years, offering diverse applications across taxa and/or tissues of interest (Metscher, 2009a; Descamps et al., 2014).

However, few studies have undertaken a rigorous assessment of stain penetration using Lugol’s iodine. And, only the cephalic region of some species of freshwater fish, reptiles, birds, and mammals have been studied using diceCT (Holliday et al., 2006, 2013; Gignac and Kley, 2014, 2018; Anderson and Maga, 2015; Clement et al., 2015; Li et al., 2016). Staining procedures (length of time in stain and stain concentration) have been found to vary significantly in different animal groups, with sample size and the mitigation of tissue shrinkage being the major drivers. To date, no marine and/or cartilaginous fish species have been examined using this method, and there are limited published data available on how diceCT can be used to investigate neuroanatomy in fish (Gignac et al., 2016; Gignac and Kley, 2018).

Here, we present a methodology for optimizing tissue contrast in the central and peripheral nervous systems of the brownbanded bamboo shark, *Chiloscyllium punctatum*, and the common goldfish, *Carassius auratus*, using diceCT. The aim of this optimization procedure is to provide suitable contrast between neural tissue and background tissue(s) of the head, thereby facilitating digital segmentation of the brain and volumetric analysis. The primary objectives of the study are to (1) determine the optimal staining time for contrast enhancement by monitoring stain uptake; (2) assess overall tissue shrinkage; and (3) report on the staining quality in the peripheral nervous system.

## Materials and Methods

### Animal Ethics Statement

*Chiloscyllium punctatum* specimens were acquired as juveniles or egg cases from an approved commercial breeding colony in Queensland, Australia, and euthanised under The University of Western Australia (UWA) Animal Ethics Approval RA/3/100/1153 prior to being donated to the present study. *Carassius auratus* specimens were acquired from local commercial vendors and euthanised under UWA Animal Ethics Approval RA/3/100/1220. This study was carried out in strict accordance with the ethics guidelines of The University of Western Australia and the Australian Code of Practice for the Care and Use of Animals for Scientific Purposes (National Health and Medical Research Council, 2013).

### Specimens

Eight specimens were used in this study: four immature individuals of the brownbanded bamboo shark *C. punctatum* (three females; one male), ranging from 25 to 36.5 cm in total length and from 45.24 to 114 g in body weight, and four immature individuals of the common goldfish *C. auratus* (undetermined sex), ranging from 8.5 to 9.5 cm in total length and 8.5–12 g in body weight (Table 1).

**Table 1:** Morphometrics of specimens and volumes of solutions used for fixation by transcardial perfusion

Species	Specimen	Sex	Maturity	BW (g)	TL (cm)	V_PB_ (ml)	V_fixative_ (ml)
*C. punctatum*	CP 1* CP 2	F F	im im	45.2 89.0	25.0 31.0	100 150	200 250
	CP 3	F	im	114.0	36.5	150	250
	CP 4	M	im	92.0	32.0	150	250
*C. auratus*	CA 1*	*un*	*un*	10.7	8.5	50	100
	CA 2	*un*	*un*	12.0	9.0	50	100
	CA 3	*un*	*un*	8.8	9.5	50	100
	CA 4	*un*	*un*	8.5	9.1	50	100

CP, C. *punctatum*; CA, C. *auratus*; *specimens used to trial the staining method before the study; *un*, unknown; i.m., immature; F, female; M, male; BW, body weight in grams; TL, total length in centimetres; V, volume in milliliters; PB, phosphate buffer; fixative, modified Karnovsky’s fixative.

### Specimen preparation

#### Fixation

All specimens were euthanized by an anesthetic overdose of tricaine methanesulfonate salt (ethyl 3-aminobenzoate methanesulfonate, MS-222; 250–500 mg/l) in seawater (*C. punctatum)* or freshwater (*C. auratus*), buffered to pH 7.2 with an equal concentration of sodium bicarbonate (or sodium hydrogen carbonate, NaHCO_3_). Morphometric data (total length, body weight, sex, and maturity) were recorded for all individuals (Table 1). All specimens were transcardially perfused with 0.1 m Sorensen’s phosphate buffer, followed by a modified Karnovsky’s fixative solution (2.5% glutaraldehyde, 1% paraformaldehyde, 4% sucrose, and 1% dimethyl sulfoxide in 0.13 m Sorensen’s phosphate buffer) using a perfusion pump (Cole-Palmer Instrument Co). The volumes of buffer and fixative used for each specimen are presented in Table 1. Following perfusion, the head of each specimen was severed posterior to the last gill slitfor *C. punctatum*, behind the gill cover for *C. auratus*, i.e. approximately between the second and third cervical vertebra, postfixed by immersion in modified Karnovsky’s fixative, and stored at 4°C for at least 10 d before further processing.

#### Staining

All specimens were stained at room temperature (22°C constant) on a plate stirrer with a solution of Lugol’s iodine (I_2_KI) comprising 1% w/v of I_2_ and 2% w/v of KI in distilled water (1 g I_2_ + 2 g KI in 100 ml of distilled water; Culling, 1963). One specimen of each species, *C. punctatum* (CP1) and *C. auratus* (CA1; see specimen abbreviations in Table 1), was used to optimize the staining and X-ray μCT scan parameters. These specimens were scanned unstained (T_0_ = 0 h) and then rescanned every 24 h after being placed in 200 ml of stain. After 24 h (T_1_ = 24 h), all the stain had been absorbed by the tissues, leaving a clear solution. Therefore, the stain solution was replaced with fresh solution after each scanning time point. It was also noted that the lenses of the eyes strongly absorbed the iodine, leading to beam hardening artefacts during the μCT scans. To avoid this artifact, the ocular lenses of all specimens were removed after the T_0_ scan by making a small incision in the limbus and severing both the suspensory ligament and the accommodatory eye muscle. Otherwise the eye cups were left in place. Based on these preliminary experiments, all remaining specimens of *C. punctatum* (*n *=* *3; CP2-4) and *C. auratus* (*n *=* *3; CA2-4) were, respectively, placed in 300 and 50 ml of stain solution at T_0_, then scanned every 48 h (*C. punctatum*) and 24 h (*C. auratus*).

### Imaging

All specimens were mounted either within a 50-ml Falcon tube (*C. punctatum*) or a 20-ml plastic syringe (*C. auratus*), in air, with PBS-soaked tissue at the base to keep the sample moist and physically stable during scanning. Specimens were scanned using an x-ray microscope (Versa 520 XRM, Zeiss) located at the Centre for Microscopy, Characterisation and Analysis (CMCA), The University of Western Australia.

To assess stain penetration, specimens were scanned using the μCT parameters presented in Table 2. For each specimen, an initial scan (scout scan) was performed, with the minimal detail needed to select adequate parameters (e.g. the desired range of sample position, field of view/voxel size, and objective) to be used in subsequent scans. Staining optimization times were assessed using reconstructed data from the subsequent scans (Table 2), using a semi-automated method (see below, Data analysis). When suitable contrast was reached across the whole brain (CNS; T_10_ for *C. punctatum*; T_4_ for *C. auratus*), a second, more detailed scan of each specimen (*n *=* *6) was undertaken, using the scan parameters in Table 2. A third, higher resolution scan of the olfactory peripheral nervous system and the forebrain was also performed on each sample using the scan parameters in Table 2, to visualize staining effectiveness.

**Table 2:** CT scanning parameters for each specimen used for the different scanning times during the staining optimisation (a.), and subsequent brain and forebrain study (b., c.)

Specimen	Voltage(kV)	Amperage(μA)	Filter(LE)	Source(mm)	Detector(mm)	Voxel(μm)	Objectivemagnification	Binning	Exposuretime (s)	Projectionsnumber	Scanningtime(min)
a. Scan at each staining time point, scout and scan parameters											
CP 1	60	5	3	–66	76	31.99	0.4×	2	1	401	30
CP 2	80	7	3	–90	42.6	23.43	0.4×	1	5	401	60
CP 3	80	7	3	–90	42.6	23.43	0.4×	1	5	401	60
CP 4	80	7	3	–90	42.6	23.43	0.4×	1	5	401	60
CA 1	60	5	2	–45	100	21.43	0.4×	2	1	401	30
CA 2	60	5	2	–45	100	21.43	0.4×	2	1	401	30
CA 3	60	5	2	–45	100	21.43	0.4×	2	1	401	30
CA 4	60	5	2	–45	100	21.43	0.4×	2	1	401	30
b. Brain scan at optimized staining time, detailed scan parameters											
CP 1	80	7	3	–66	76	31.99	0.4×	1	5	2501	360
CP 2	80	7	3	–90	42.6	23.43	0.4×	1	5	2501	360
CP 3	80	7	3	–90	42.6	23.43	0.4×	1	5	2501	360
CP 4	80	7	3	–90	42.6	23.43	0.4×	1	5	2501	360
CA 2	60	5	2	–45	100	21.43	0.4×	2	2	2501	180
CA 3	60	5	2	–45	100	21.43	0.4×	2	2	2501	180
CA 4	60	5	2	–45	100	21.43	0.4×	2	2	2501	180
c. Forebrain scan at optimized staining time, higher resolution scan parameters											
CP 1	80	7	3	–45	103	11	0.4×	1	5	2501	380
CP 2	80	7	3	–54	103	11.88	0.4×	1	5	2501	393
CP 3	80	7	3	–54	103	11.88	0.4×	1	5	2501	393
CP 4	80	7	3	–54	103	11.88	0.4×	1	5	2501	393
CA 2	60	5	2	–25	145	10	0.4×	2	3	2501	180
CA 3	60	5	2	–25	145	10	0.4×	2	3	2501	180
CA 4	60	5	2	–25	145	10	0.4×	2	3	2501	180

For specimen abbreviations, see Table 1.

**Table 3:** Scaling parameters used to standardise data reconstructions for the scans using the parameters in Tables 2a and 2b: byte scaling

CT scale filter	Beamhardeningconstant (BHC)	Min	Max
CP-401 CP-2501 CA-401 CA-2501	0.05 0.05 0 0	–0.0002696328 0.0001733653 –0.0002696328 0.0001733653	0.0948172 0.7160122 0.0948172 0.7160122

CP, *C. punctatum*; CA, *C. auratus*; 401, number of projections used for scans utilizing the CT parameters in Table 2; 2501, number of projections used for scans utilizing the CT parameters in Table 2.

### Data processing

#### Tomographic reconstruction

The projection data from each scan was reconstructed using XRMReconstructor (11.1.5707.17179, Zeiss) to obtain a spatial volume comprising a 3D array of cubic voxels with intensities standardized to Hounsfield units (HU). Scaling to radiodensity in HU was performed using linear attenuation coefficients derived from scans of water and air phantoms, acquired under identical conditions to those listed in Table 2 for the stain optimization and brain studies, following the protocol of Candell (2009). Reconstructions followed a standard automatic center shift correction in addition to the parameters listed in Table 3. A default reconstruction filter of σ 0.7 (smoothing filter) was applied to all data. Data from forebrain scans were reconstructed without scaling, as there was no intention to compare stain uptake between time points in subsequent studies.

#### Spatial co-registration of image volumes and volume-of-interest cropping

To facilitate spatial comparisons of staining over time, each time series of image volumes (single specimen scanned before staining and at successive time points thereafter) was spatially aligned or co-registered to its T_0_ volume based on fixed anatomic features. The selected anatomic features were the skin surface of the head for *C. punctatum* and the head bones and brain case for *C. auratus*. Alignment was performed using Avizo (Standard v 9.2.0, Thermo-Fisher Scientific) in two steps (see Tables 4, 5 for *C. punctatum* and *C. auratus*, respectively). First, the T_0_ volume was visualized as a volume rendering and manually rotated and translated (Transform Editor) to align the heads such that the dorso-ventral and antero-posterior anatomic orientation matched the *z*- and *x*-axes, respectively. The T_0_ volume was then resampled according to these new coordinates (resample transformed image module). Second, the volumes from the remaining time points were registered in turn to this reference T_0_ volume (register images module) using a rigid body transformation (translations and rotations only). For *C. punctatum*, the registration of the heads was performed on the thresholded and filled (3D hole-filling) versions of the volumes using cross-correlation. For *C. auratus*, the registration of the brain cases was performed using normalized mutual information with a subset of intensity values corresponding to the head bones. Finally, each of the aligned volumes were cropped to obtain a volume-of-interest defined by the head boundaries (skin surface) and the caudal boundary of the brain, i.e., at the level of the first set of cervical spinal nerves (Northcutt, 1977, 1978). Following registration, all data were resampled according to these new coordinates (resample transformed image module). This facilitated our navigation to the same positions in each specimen. Detailed workflows are provided in Tables 4, 5, for *C. punctatum* and *C. auratus*, respectively.

**Table 4:** Workflow used to digitally process all shark specimens using Avizo (standard 9.2.0)

Step	Module/operation	Subparameters/inputs	Justifications
**1**	Import the datasets (i.e., each .txm file) for each stain time of a specimen in the project vView	• Edit: click preferences• Units tab: tick use and choose correct units	To ensure each dataset’s voxel size and unit measure are correct
**2**	Save project as	• File: save as project• Type: Avizo pack & go• Save onto local disk	To save the project now the datasets to work with are imported

**Start with CP1-T0**			

**3**	Volume rendering	• Toolbar: Orthographic view	To quickly first check your data and make sure you are working with the orthographic view and **not the perspective view**
**4**	Extract subvolume	• Right click dataset• Search: Extract subvolume	To crop the data cube to a smaller size (remove the empty space around the head) for faster computation
**5**	Global axes	• View: tick Global Axes	To visualize three-dimensional axes
**6**	Transform editor	• Left click on the green module created in step 4• In the Properties window toolbar: click on Transform Editor• Manually align head to global axes by pressing Shift while moving with the Interact cursor, based on chosen landmarks using Trackball tool in main window toolbar	To align head to a chosen standard position to use relative to global axes
**7**	Resample transformed image	• Right click the green module from step 4• Search: Resample transformed image• Interpolation: Lanczos• Mode: extended• Keep other defaults	To re-slice each sample according to the global axis and set alignments
**8**	Extract subvolume	• Right click dataset• Search: Extract subvolume	To crop the data cube again to a smaller size including only the head as the bounding box is bigger after resampling the transformed image
**9**	Resample	• Filter: Lanczos• Mode: voxel size• Resolution: divided by three for *x*, *y*, and *z* values (26.14 → 78.43 μm)	To down sample; in this instance, to a third of the initial resolution, to fasten the computations to come
**10**	Edit new label field	• Right click dataset created in step 9• Search: Edit new label field• Threshold tool: select all head and assign to a label• Select the label newly assigned• Toolbar: Selection; Fill; All slices• Repeat filling (previous bullet point) for all panels in the Segmentation Editor, XY, YZ and XZ	To select the head and fill the inner volume

**Continue with CP1-T1**			

**11**	Resample	• Reference: T0.resampled• Filter: Lanczos• Mode: voxel size	To down sample using the resolution of T0 in step 3, so the computation in the Segmentation Editor is faster
**12**	Edit new label field	• Threshold tool: select all head and assign to a label• Select the label newly assigned• Toolbar: select X; Fill selection; select slice (not 3d volume); fill all slices in the three directions *x*, *y*, and *z* in the four panes window	To select the head; same as step 10 but in sample at T1
**13**	Register images	• Right click green module CP1-T1.txm (dataset)• Search: Register images• In Properties window:• Model: CP1-T1.labels• Reference: CP1-T0.labels• Transformation: Rigid• Metric: label difference• Keep other defaults• Prealign: click Align lefts, then Align principal axes• Apply	To register T1-labeled head with T0-labeled head
**14**	Copy transformation	• Data: CP1-T1.txm• Reference: CP1-T1.labels	To use the previous registration pathway to register the actual dataset
**15**	Resample transformed image	• Right click green module CP1-T1.txm (dataset)• Search: Resample transformed image• Data: CP1-T1.txm• Interpolation: Lanczos• Mode: cropped• Apply	To re-slice according the alignment of reference T_0_

**REPEAT steps 11–15 for each other staining time T_i_**.			
**Then, do the following for all staining times**			

**16**	ROI box	• Right click on each green module created in step 10• Search: ROI box• Create for each sample• Shift select each of the ROI box• Click on the Connection Editor on all of them in the Properties window• Click and drag to link all the Minimum [unit] and Maximum [unit] from the reference ROI Box to the next	To select the same area including only the head for all samples
**17**	Crop editor	• Left click on the green module created in step 10• In the Properties window toolbar: click on Crop Editor• In the small window that opens; Crop list: tick “Use ROI list”• Resolution mode: bounding box	To crop all ROI boxes to the same volume
**18**	Orthoslice	• Right click on each green module created in step 15• Search: Orthoslice to create one	To check all orthoslices are matching in all three views (XY, XZ, and YZ) in the main display window
**19**	Resample	• Data: green module obtained in step 15• Reference: NO SOURCE• Filter: Lanczos• Mode: voxel size• Voxel size [units]: doubled	To down sample by half and speed up filtering computations in the next step
**20**	Non-local means filter	• Right click on each green module created in step 15• Search: Non-local means filter• Keep all defaults but make Filter: XY planes is selected• Apply	To smooth or reduce noise
**21**	Orthoslice	• Right click on each green module created in step 20• Create four orthoslices for each• Set Colormap to standardized range for all	To position the four sampling slices in transversal view at the position of interest in the brain
**22**	Line probe	• Right click on each green module created in step 20• Search: Line probe and create four probes• Enter Slice number corresponding to slices chosen in step 21• Sampling options: number of samples 500	To position the four lines probes corresponding to each orthoslice using the same coordinates as the reference line probe chosen for all times

**Select each orthoslice and corresponding line probe sequentially to download the data**.			

**Table 5:** Workflow used to digitally process all goldfish specimens using Avizo (standard 9.2.0)

Step	Module/operation	Subparameters/inputs	Justifications
**1**	Import the datasets (i.e., each .txm file) for each stain time of a specimen in the Project View	• Edit: click preferences• Units tab: tick use and choose correct units	To ensure each dataset’s voxel size and unit measure are correct
**2**	Save project as	• File: save as project• Type: Avizo pack & go (.am format)• Save onto local disk	To save the project now the datasets to work with are imported

**Start with GF1-T0**			

**3**	Volume rendering	• Right click green dataset• Search: Volume rendering• Toolbar: Orthographic view	To quickly first check your data and make sure you are working with the orthographic view and **not the perspective view**
**4**	Extract subvolume	• Right click dataset• Search: Extract subvolume	To crop the data cube to a smaller size (remove the empty space around the head) for faster computation
**5**	Global axes	• View: tick Global axes	
**6**	Transform editor	• Left click on the green module created in step 4• In the Properties window toolbar: click on Transform editor• Manually align head to global axes by pressing Shift while moving with the Interact cursor, based on chosen landmarks using Trackball tool in main window toolbar	To align head to a chosen standard position to use relative to global axes
**7**	Resample transformed image	• Right click the green module from step 4• Search: Resample transformed image• Interpolation: Lanczos• Mode: extended• Keep other defaults	To re-slice each sample according to the global axis and set alignments
**8**	Extract subvolume	• Right click dataset• Search: Extract subvolume	To crop the data cube again to a smaller size including only the head as the bounding box is bigger after resampling the transformed image

**Continue with GF1-T1**			

**9**	Register images	• Right click green module GF21-T1.txm• Search: Register images• In Properties window:• Model: GF1-T1.labels• Reference: GF1-T0.labels• Keep all defaults below• Transformation: Rigid• Metric: Normalized mutual information• Prealign: click Align lefts, then Align principal axes• Click Apply	To overlay GF1-T1 on GF21-T0
**10**	Resample transformed image	• Right click green module GF21-T1.txm• Search: Resample transformed image• Interpolation: Lanczos• Mode: extended• Keep other defaults	To re-slice according the alignment of reference T_0_

**Repeat steps 9–10 for each other staining time T_i_ and then, do the following for all staining times**			

**11**	ROI box	• Right click on each green module created in step 10• Search: ROI box• Create for each sample• Shift select each of the ROI box• Click on the Connection Editor on all of them in the Properties window• Click and drag to link all the Minimum [unit] and Maximum [unit] from the reference ROI box to the next	To select the same area including only the head for all samples
**12**	Crop editor	• Left click on the green module created in step 10• In the Properties window toolbar: click on Crop Editor• In the small window that opens; Crop list: tick “Use ROI list”• Resolution mode: bounding box	To crop all ROI boxes to the same volume
**13**	Orthoslice	• Right click on each green module created in step 10• Search: Orthoslice to create one	To check all orthoslices are matching in all three views (XY, XZ, and YZ) in the main display window
**14**	Non-local means filter	• Right click on each green module created in step 10• Search: Non-local means filter• Keep all defaults but make sure Filter: XY planes is selected• Apply	To smooth or reduce noise
**15**	Orthoslice	• Right click on each green module created in step 14• Create four orthoslices for each• Set Colormap to standardized range for all	To position the four sampling slices in transversal view at the position of interest in the brain
**16**	Line probe	• Right click on each green module created in step 14• Search: Line probe and create four probes• Enter Slice number corresponding to slices chosen in step 15• Sampling options: number of samples 500	To position the four lines probes corresponding to each orthoslice using the same coordinates as the reference line probe chosen for all times

**Select each orthoslice and corresponding line probe sequentially to download the data**			

**Table 6:** Percentage of tissue shrinkage for the different brain areas sampled in each specimen, and the average levels of shrinkage per area and for the whole brain (*n *=* *3 specimens per species)

	Tissue shrinkage (%)									
	*C. punctatum*					*C. auratus*				
	CP2	CP3	CP4	Mean	±SD	CA2	CA3	CA4	Mean	±SD
OBs	13.20	7.61	21.48	14.09	6.98	11.61	9.13	5.75	8.83	2.94
Tel	11.04	11.89	14.63	12.52	1.88	14.62	17.26	27.76	19.88	6.95
Cer	12.74	12.01	7.44	10.73	2.87	24.90	25.57	20.13	23.53	2.96
Med	18.79	10.24	21.36	16.79	5.82	13.68	16.39	26.42	18.83	6.71
Overall				13.54	4.75				17.77	7.25

CP, *C. punctatum*; CA, *C. auratus*; SD, standard deviation; OBs, olfactory bulbs; Tel, telencephalon measured on slice 2; Cer, cerebellum; Med, medulla oblongata.

#### Sampling intensity values

**Figure 1.**
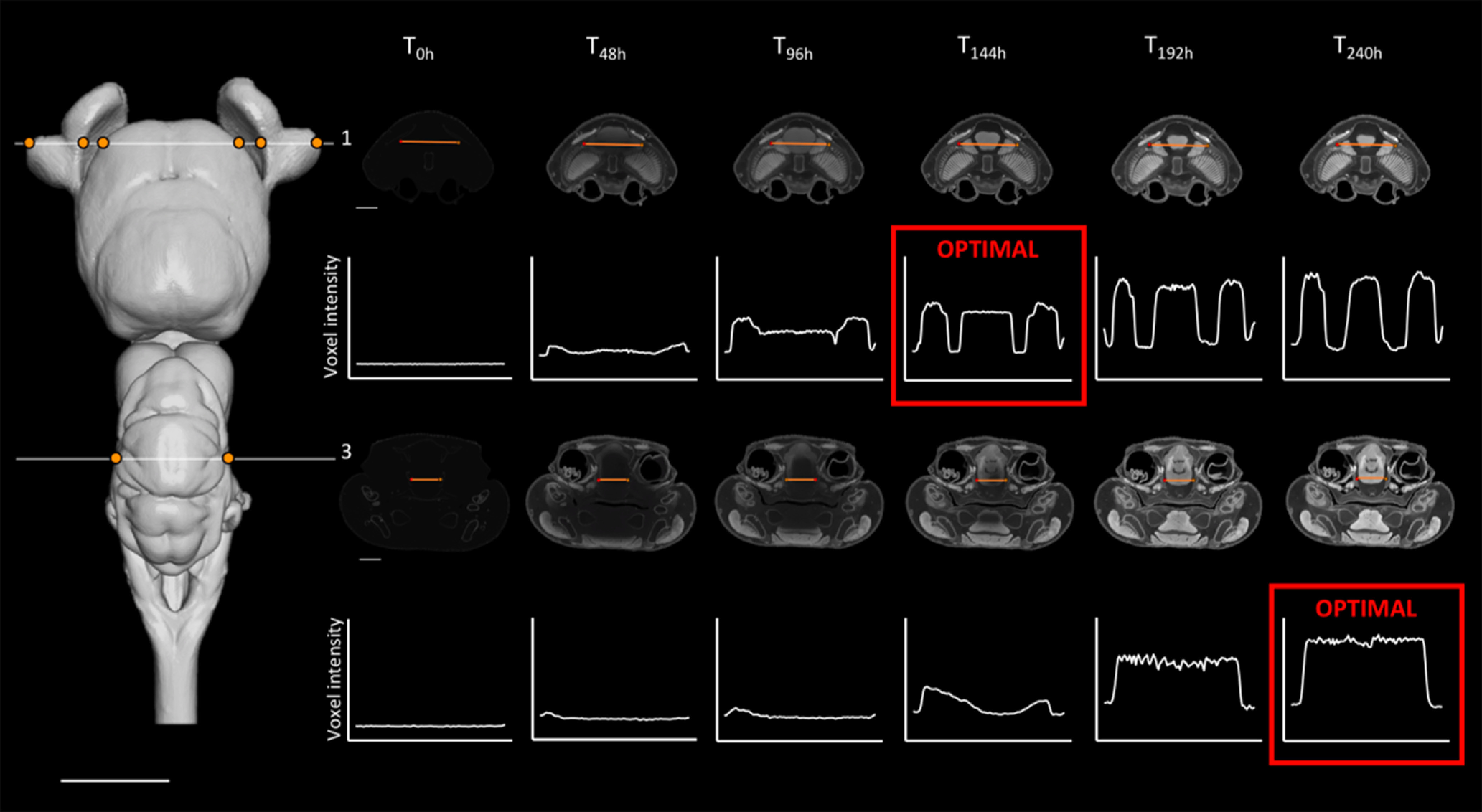
Optimal staining times (in red; see definition in the text) for individual slices (indicated in red) for two of the four slices sampled (numbers 1 and 3) across the brain of *C. punctatum*. Left, Dorsal view of the segmented brain of *C. punctatum*, showing the positions of slices 1 and 3 in white. Right, Positions of the line profiles within each slice (orange lines), across staining time points, and plots showing the voxel intensity values corresponding to the line profile at each time point. The intensity range for all orthoslices presented was set at 0–20,000 HU. Scale bars = 5 mm.

**Figure 2.**
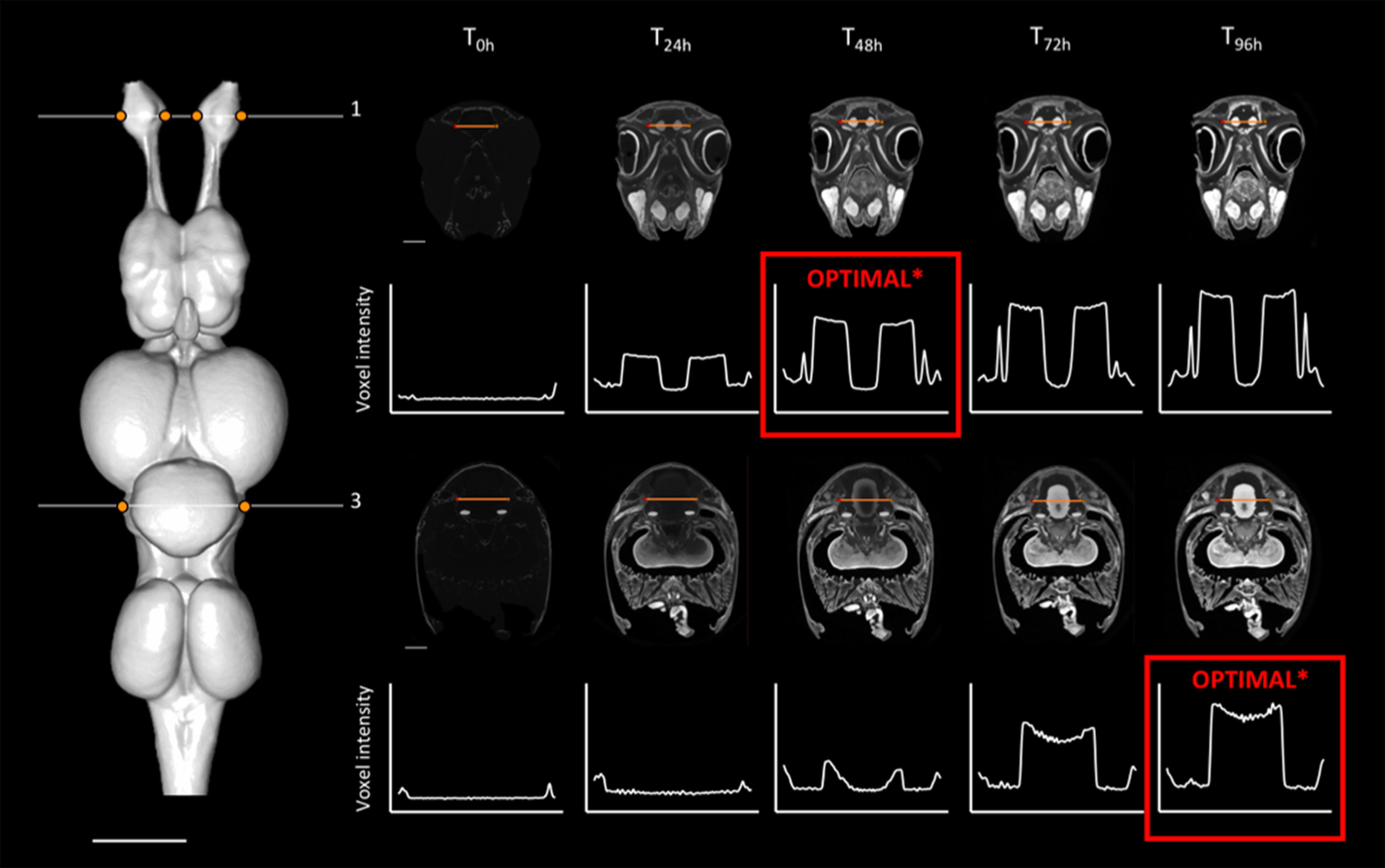
Optimal staining times (in red; see definition in the text) for two of the four slices sampled (numbers 1 and 3) across the brain of *C. auratus*. Left, Dorsal view of the segmented brain of *C. auratus*, showing the positions of slices 1 and 3 in white. Right, Positions of the line profiles within each slice (orange lines), across staining time points, and plots showing the voxel intensity values corresponding to the line profile at each time point. The intensity range for all orthoslices presented was set at 0–40,000 HU. *, Given the difference in brain tissue composition (myelinated and unmyelinated neuronal fibers) found in the represented regions of this species, the intratissue variation of intensity values in the brain (P3) still shows staining heterogeneity (non-horizontal curve). Scale bars = 2 mm.

**Figure 3.**
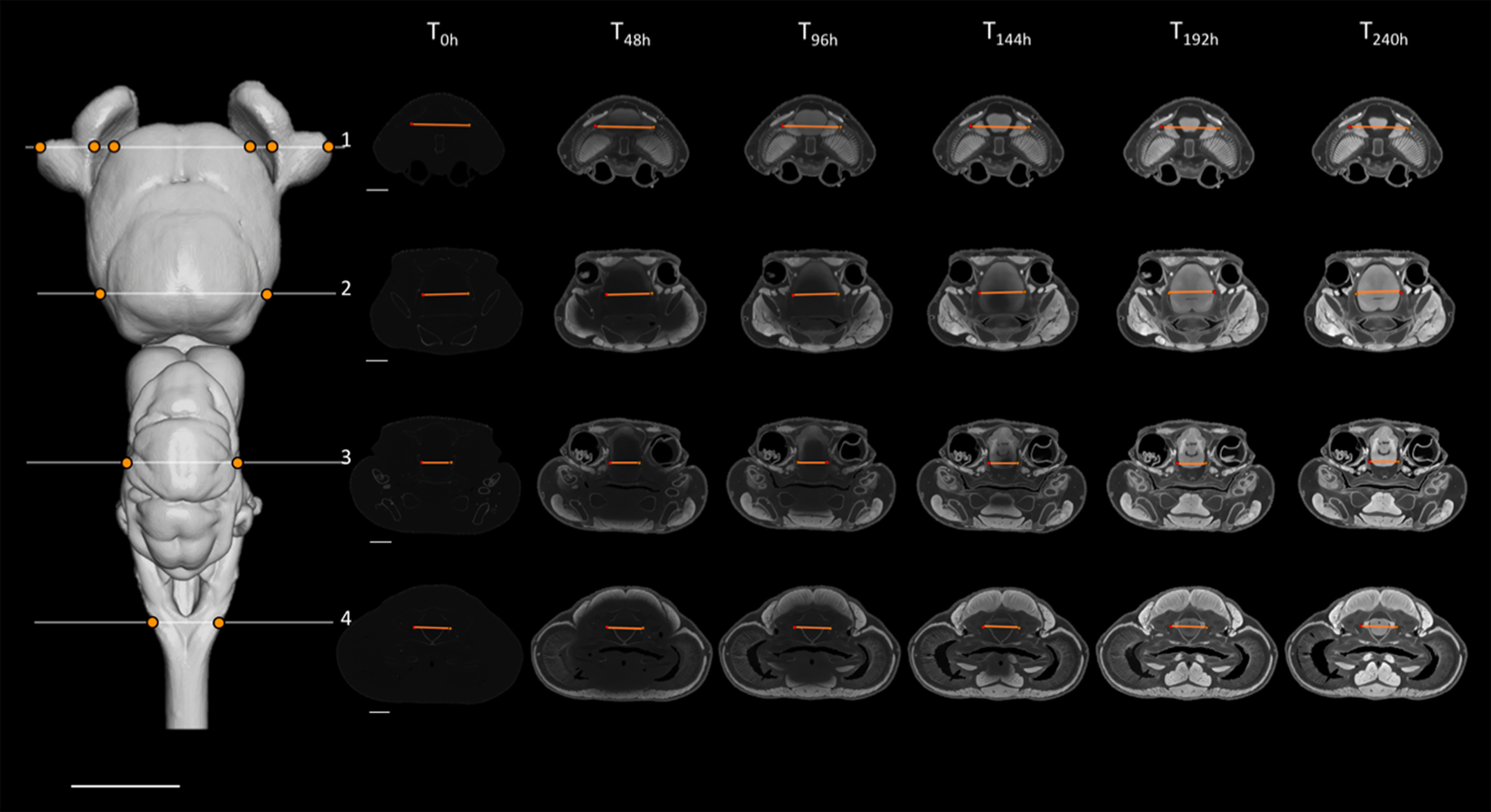
Sampling design used for the present staining optimization study, showing the segmented brain of *C. punctatum* in dorsal view (left) and the positions of the four slices sampled (numbered 1–4 in white) and the positions of the line profiles within each slice (orange lines), sampled consistently across staining time points (right). Slices on the right show frontal transections corresponding to the brain slice number on which the peak analysis was conducted and statistics of the voxel intensity values were monitored across the six time points (T_0h_–T_240h_), for all the four line profiles sampled (using the tissue edges; orange points). Intensity range for all orthoslices presented was set between 0 and 20,000 HU. Scale bars = 5 mm.

**Figure 4.**
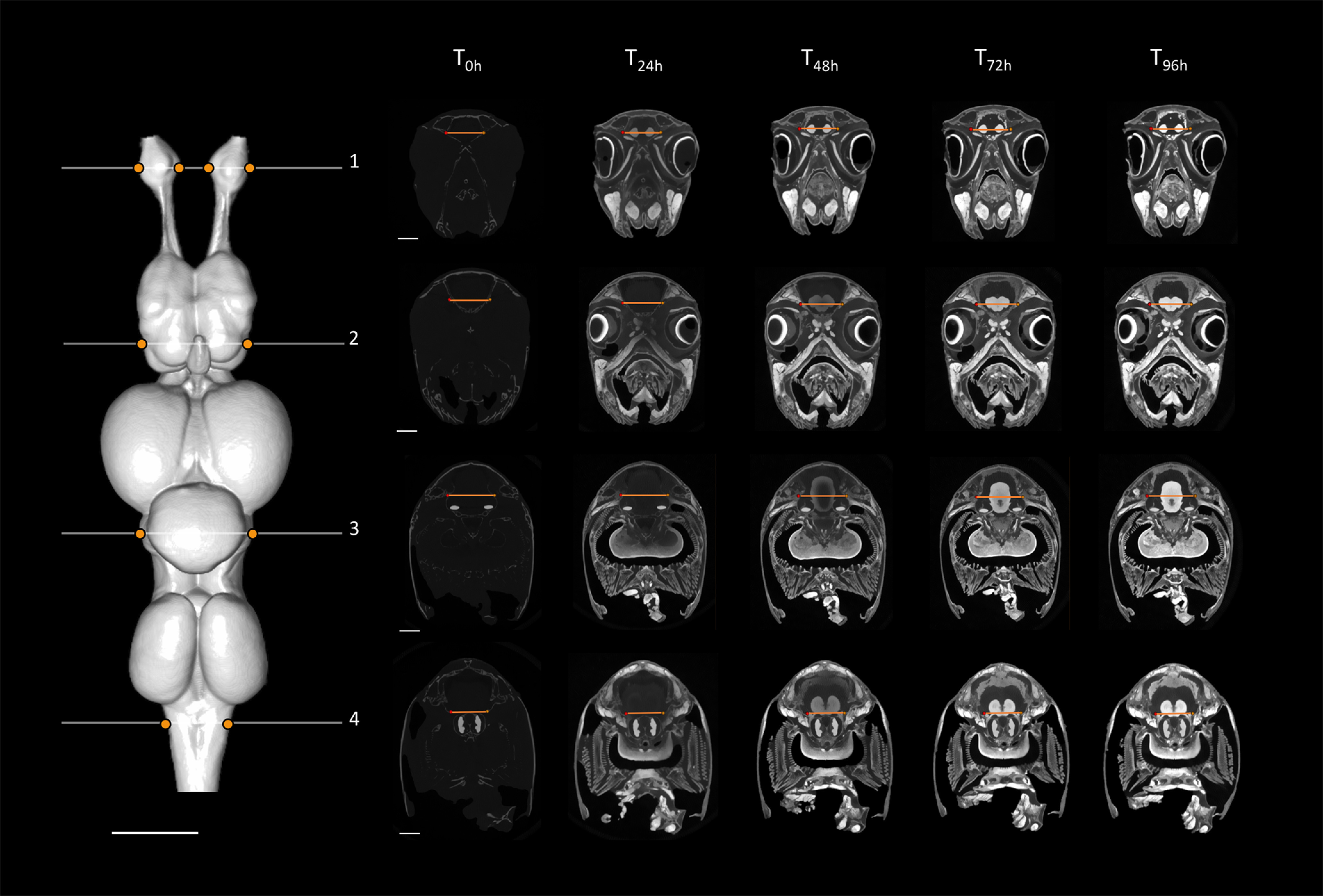
Sampling design used for the present staining optimization study, showing the segmented brain of *C. auratus* in dorsal view (left) and the positions of the four slices sampled (numbered 1–4 in white) and the positions of the line profiles within each slice (orange lines), sampled consistently across staining time points (right). Slices on the right show frontal transections corresponding to the brain slice number on which the peak analysis was conducted and statistics of the voxel intensity values were monitored across the six time points (T_0h_–T_96h_), for all the four line profiles sampled (using the tissue edges; orange points). Intensity range for all orthoslices presented was set between 0 and 40,000 HU. Scale bars = 2 mm.

For each specimen, we selected four transverse planes (orthoslices perpendicular to the anterior-posterior axis) at similar positions across the brains of the aligned volumes (Figs. 1, 2; further details in Fig. 3 for *C. punctatum* and Fig. 4 for *C. auratus*). The positions of these planes were chosen to include several types of brain tissue from the forebrain to the caudal boundary of the hindbrain. The specific planes assessed were: (1) across the widest plane of the olfactory bulbs (OBs); (2) the widest plane of the telencephalon dorso-ventrally (Tel); (3) anterior to cranial nerve VII, just anterior to the lobus lineae laterali (or *recessus lateralis ventriculi quarti*) in the cerebellum (Cer); and (4) posterior to the vagus nerve (cranial nerve X) in the caudal area of the medulla oblongata, rostral to the first set of spinal nerves (Med). Horizontal (left-to-right) line probes were then placed on each of the four slices (2D images) using chosen landmarks for each species, avoiding the ventricles (Figs. 1-4). To ensure the same area of the brain was sampled at each time point, the same anatomic coordinates were used. Although the registration step resulted in a close alignment of the slice position for each staining time point, minor alterations in anatomy, arising primarily from tissue shrinkage, meant that a slight manual adjustment was needed to ensure the same region was chosen for analysis. The intensity profiles along each of these line probes was recorded (*n *=* *500 sample points).

### Data analysis

#### Extracting slice-based parameters of interest

**Figure 5.**
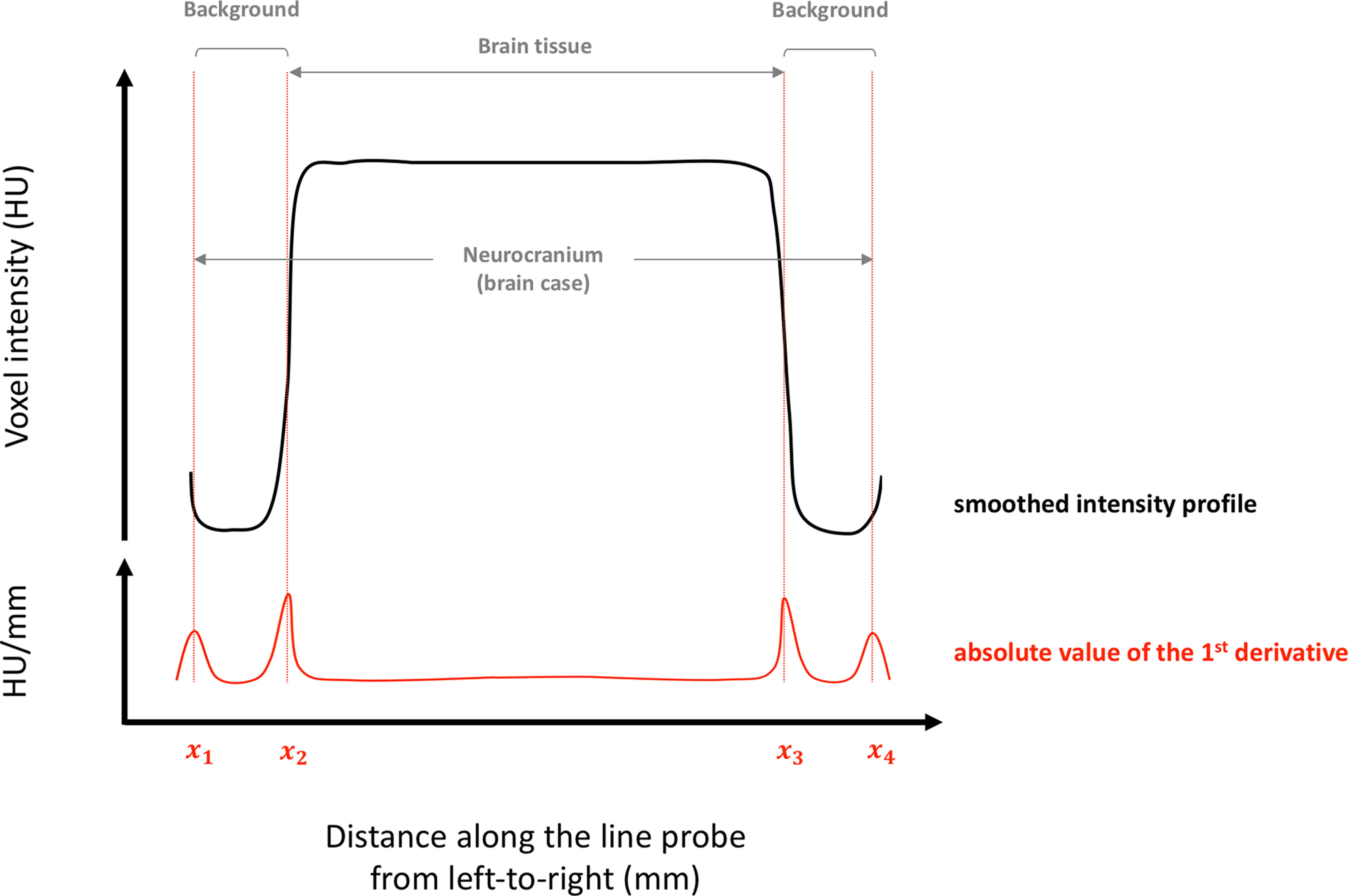
Diagram illustrating the peak analysis undertaken to extract slice-based parameters for a sample slice. P1 is computed for each tissue edge. Each tissue edge corresponds to a peak in the absolute value of the first derivative of the smoothed profile (positions $x_{1},x_{2},x_{3},x_{4}$). The value of the parameter is defined to be the height of the peak, which is a measure of edge strength. P2 and P3 are computed from the smoothed intensity profile. P2 is the difference of mean intensity values between brain tissue and background (endocast); i.e., the mean of the intensity values in the interval $\left(x_{2},x_{3}\right)$ minus the mean of the intensity values taken from the intervals $\left(x_{1},x_{2}\right)$ and $\left(x_{3},x_{4}\right)$. P3 is the interquartile range of intensity values within brain tissue; i.e., values in the interval $\left(x_{2},x_{3}\right)$. P1 and P2 are both proxies for brain contrast levels intertissue. P3 is a proxy for staining homogeneity intratissue. P4 is the brain tissue width (in mm); i.e., $x_{3}-x_{2}$.

For each intensity profile (intensity values are in HU), we performed a peak analysis using a custom-written R script (see Extended Data 1). The script smooths the intensity profile using a cubic smoothing spline, computes the first derivative (which describes the instantaneous rate of change of intensity at each point), and locates its significant positive and negative peaks. The locations of these peaks correspond to the edges between intervals of differing intensities in the intensity profile (i.e., positions of tissue edges). The absolute value of the peak height is a measure of the strength of the corresponding edge. The script automatically selects pairs of peaks about the midpoint of the line probe. It allows the user to interactively adjust the selection, where the chosen peaks do not coincide with the desired tissue edges. This can occur, for example, when the stain has not yet reached the central portions of a tissue, where a trough may exist within the intensity profile. The program provides the edge position (*x* value representing the distance from the start of the line probe, from left to right, in mm) and edge strength values, as well as statistics of the intensity values (mean, median, standard deviation, interquartile range) of the smoothed profile between each pair of edges (for an illustration of the peak analysis used to extract the computed measures from the raw data, see Fig. 5, and the extracted data in Extended Data 2).

10.1523/ENEURO.0076-20.2020.ed1 Extended Data 1 R code used for the peak analysis, to extract the data required for analysis. Download Extended Data 1, ZIP file.

10.1523/ENEURO.0076-20.2020.ed2 Extended Data 2 CSV table of the data extracted using the code provided in Extended Data 1. Download Extended Data 2, CSV file.

Staining was assessed by monitoring three parameters: (1) the degree of contrast at the edges of the brain tissue [parameter 1 (P1), edge strength values]; (2) the differences of mean intensity values between brain tissues and background (P2, inner brain case surrounding the brain tissues), both considered as proxies for brain contrast levels; and (3) the staining homogeneity within the tissue (P3, interquartile range of intensity values for brain tissue, including the OBs; Fig. 5). For P1, edge strength values were collected for all brain tissue edges on each slice, at each time point; i.e., 12 values in *C. punctatum* (six values from slice number 1 and two values from slices 2–4), and 10 values in *C. auratus* (four values from slice number 1 and two values from slices 2–4; Figs. 1-4, orange points). For P2, mean intensity values of the (smoothed) intensity profile between each pair of edges corresponding to brain tissues, excluding the vagus nerve X (three values on slice number 1, one value for slices 2–4 in *C. punctatum*; two values on slice number 1 and one value for slices 2–4 in *C. auratus*), were subtracted from the mean intensity values between each pair of edges corresponding to the background or brain case (four values on slice number 1, two values for slices 2–3, and four values on slice number 4 in *C. punctatum*; three values on slice number 1, two values for slices 2–3, and four values on slice number 4 in *C. auratus*), for each slice, at each time point. For P3, the interquartile range values of the (smoothed) intensity profile between each pair of edges corresponding to brain tissues, excluding the vagus nerve X, were used.

Additionally, tissue linear shrinkage was assessed in all specimens by measuring brain tissue width (in mm), on each of the four slices, across staining time points. Specifically, the distance between pairs of edges corresponding to several brain tissues, was extracted from the raw data (*x* values on the line probes) by subtracting the lower from the higher *x* value (edge position). To determine the percentage of linear shrinkage for each brain region (i.e., sampled on each slice: OBs on slice number 1, Tel on slice number 2, Cer on slice number 3, and posterior extent of the Med on slice number 4), we calculated:

$$\frac{\text{(starting width - end width)}}{\text{starting width}}\times 100\%$$

In *C. punctatum*, the telencephalon width on slice number 1 (Tel1) was not appropriate to use, as the region measured corresponded to the frontal part of this brain region, where shrinkage occurred both transversally and rostro-caudally. Therefore, we excluded the measurements of shrinkage from Tel1 in *C. punctatum*, to avoid introducing any bias and overestimating the percentage of shrinkage.

#### Computing whole-brain parameters

To present the data on the whole brain, the raw values collected above for each parameter, including tissue width, were averaged per slice (when there was more than one value per slice) and across all four slices at each time point for each specimen (*n *=* *3 specimens per species). We also calculated the mean percentage of shrinkage for the different brain areas, across specimens, and the overall mean percentage of shrinkage observed in the brain across all four areas. All analyses were conducted in R version 3.5.3 (R Core Team, 2019) and plots created using the package ggplot2 version 3.1.0 (Wickham et al., 2018).

#### Qualitative description of staining in the peripheral nervous system

Stain uptake was qualitatively observed in specific brain regions using the filtered (non-local means filter) volume rendered data from higher resolution scans (acquired under scanning parameters in Table 2c). Specifically, four peripheral sensory systems were examined in the anterior region of the head of *C. punctatum*, i.e., the olfactory, electrosensory, mechanosensory (lateral line), and non-image forming visual (pineal organ, involved in the regulation of circadian rhythms) systems. The structures examined included the olfactory organs (rosettes), olfactory nerves and bulbs, the ampullary pores and ampullae of Lorenzini in the rostral ampullary cluster, the lateral line pores and canals, and the pineal gland, as well as their innervation, when visible in the field of view. In *C. auratus*, structures within the mechanosensory, olfactory and non-image forming visual systems, as above (except for electroreception, not present in *C. auratus*), were examined in the field of view of the corresponding higher resolution scans (Table 2c).

### Data/code accessibility

The code described in this paper, developed by A.M. in R (R Core Team, 2019), is freely available online at https://github.com/VCA7997/CT-fish-brain.git. The code is available as Extended Data 1. The data used for analysis were extracted from the raw volume data using the given code and are available as Extended Data 2. The results of this study were obtained using the OS X El Capitan version 10.11.6 (15G22010) operating system on a MacBook Pro.

## Results

### Optimal staining times

The overall objective of the study was to facilitate the semi-automated segmentation of the whole brain in both fish species. To achieve this objective, a stepwise assessment of staining was undertaken to determine optimal staining using the line profile parameters (P1, P2, and P3, as defined above, Extracting slice-based parameters of interest; Fig. 5). Optimal staining is with respect to two constraints: limited scan time and the mitigation of tissue shrinkage.

**Figure 6.**
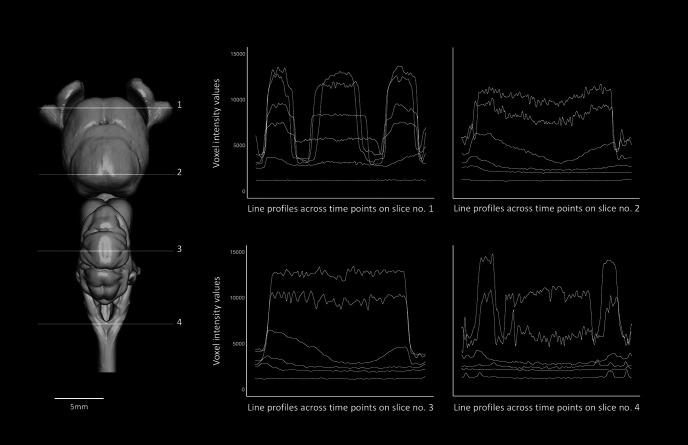
Dorsal view of the segmented brain of *C. punctatum* (left), showing the four brain slices (numbers 1–4) from which line profiles were sampled across time points (compare Fig. 3). The plots (right), show the voxel intensity values monitored across the six time points (T_0h_–T_240h_), every 48 h, for all the four line profiles sampled.

**Figure 7.**
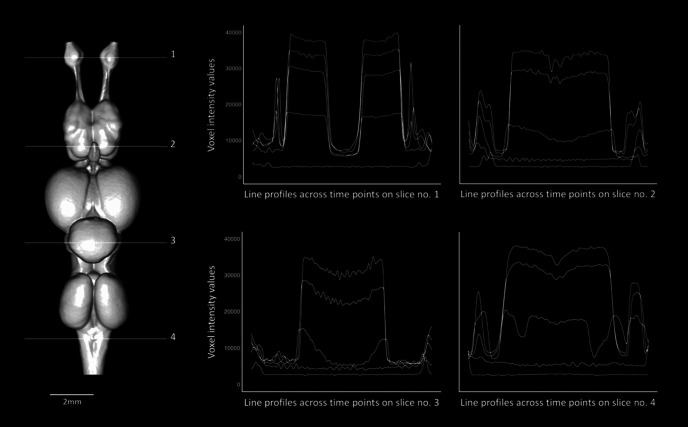
Dorsal view of the segmented brain of *C. auratus* (left), showing the four brain slices (numbers 1–4) from which line profiles were sampled across time points (compare Fig. 3*B*). The plots (right) show the voxel intensity values monitored across the six time points (T_0h_–T_96h_), every 24 h, for all the four line profiles sampled.

For individual slices, optimal staining was determined by (1) a visual assessment to select the set of time points for which a clear P1 (peak intensity) response across the line profile exists for each tissue edge, followed by (2) the selection of the time point for which P3 is the minimum value (homogeneity) within the sub-set of time points chosen in step 1; in the case where multiple candidates have similar P3 values, we chose the time point with the largest P2 (intensity difference intertissue). As expected, stain penetration progressed via a gradual ingress through the skin and through the body cavities, including the nasal passages, the mouth, and caudal head boundary (i.e., where the head was cut off from the body). Therefore, the stain penetrated faster in certain regions compared with others. This is demonstrated by differential intensity profiles between slices. For instance, homogeneous staining in the brain (horizontal intensity curve intra-tissue) was attained in the most anterior region at earlier time points than in the most caudal regions (Figs. 1, 2; further details in Figs. 3, 6 for *C. punctatum* and Figs. 4, 7 for *C. auratus*). The line profile data show that optimal staining intensity differed across the brain of each species (Figs. 1, 2). In *C. punctatum*, the line profile sampling across the olfactory bulbs (OBs) and the anterior extent of the telencephalon on slice number 1 (Tel1) suggested overstaining (convex intensity curve intratissue) in the telencephalon at the last time point; in contrast, the line profile sampling across the middle part of the cerebellum on slice number 3 (Cer) indicated an optimal staining intensity at the last time point (Fig. 1). Conversely, the larger part of the telencephalon on slice number 2 (Tel) showed that the region remained understained (concave intensity curve intra-tissue) at the last time point (Fig. 6). Moreover, the back of the brain (posterior extent of the medulla oblongata) monitored by the line profile on slice number 4 (Med) only became visible at the last time point (Fig. 6). In *C. auratus*, the line profile sampling across the OBs on slice 1 revealed an increasing staining intensity across time points, in addition to an increased variation in intensities within the tissue (decreased staining homogeneity intratissue) from time point T_48h_ (Fig. 2). The widest region of the telencephalon sampled by the line profile on slice number 2 showed that the region reached an optimal staining intensity (an almost horizontal intensity curve and homogeneous staining) at time point T_72h_ (Fig. 7). However, the line profile sampling across the cerebellum, just anterior to cranial nerve VII on slice number 3, appeared still understained at the last time point but with lesser variations in the range of intensity values (Fig. 2), possibly due to the presence of different levels of myelination. Furthermore, the posterior extent of the medulla oblongata monitored by the line profile on slice number 4 indicated that the staining was optimal (an almost horizontal intensity curve and more homogeneous staining) at the last time point, for the different types of neural tissues (nerves and brain matter) present in this area (Fig. 7).

**Figure 8.**
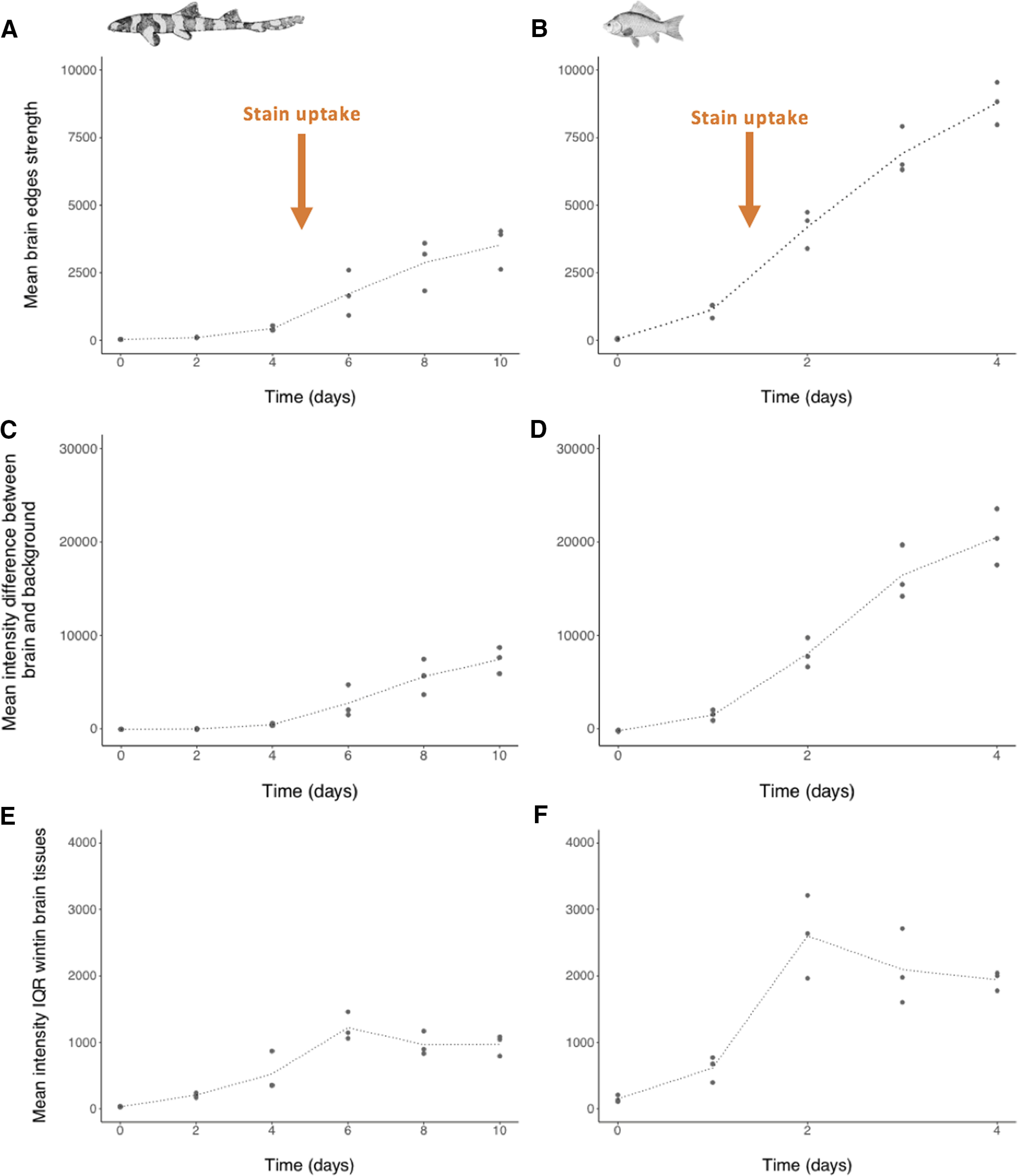
Plots of the three parameters used to monitor stain uptake in the brain from three specimens of *C. punctatum* (***A***, ***C***, ***E***) and *C. auratus* (***B***, ***D***, ***F***). ***A***, ***B***, Mean brain tissues edge strength values. ***C***, ***D***, Mean intensity difference between brain tissues and background, or inner brain case (excluding the vagus nerve X on slice 4). ***E***, ***F***, Mean intensity interquartile range (IQR) in the brain tissues (excluding the vagus nerve X on slice 4). Each data point represents the average of raw values collected from all brain tissues sampled across all slices for one specimen. Black dotted lines join the mean value from the three replicate points per time point, to illustrate the trend over time.

For the whole brain, optimality was determined based on the pooled mean for each parameter (P1–P3) across all the four slices (Fig. 8). In all cases, and for both species, the optimal staining time point was unambiguously the last staining time point; where mean P1 was a maximum (Fig. 8*A*,*B*), mean P2 was a maximum (Fig. 8*C*,*D*), and mean P3, following the onset of stain uptake, was a minimum (Fig. 8*E*,*F*). For the whole brain, the optimal staining times were thus found to be 10 d for *C. punctatum* and 4 d for *C. auratus* (Fig. 8).

#### Monitoring contrast levels for the whole brain

For the whole brain (*n *=* *4 slices pooled), mean P1 and mean P2 indicated that, in unstained samples (T_0_), there was no contrast observed between brain tissues and background (brain case) for both model species (Fig. 8). At the edges of the brain tissues, contrast increased in both species, i.e., from day 0 to 10 in *C. punctatum* (amplitude ranged from 0.77 to 7098.24), and from day 0 to 4 in *C. auratus* (amplitude ranged from 2.93 to 12,849.75; Fig. 8*A*,*B*). In *C. punctatum*, the amplitudes remained close to zero until day 4, then increased markedly between days 4 and 6, before starting to plateau from day 6 (Fig. 8*A*). In *C. auratus*, the amplitudes were close to zero at T_0_, and increased as soon as they were placed in the stain, suggesting faster uptake of stain compared with *C. punctatum* specimens. The values increased markedly between days 1 and 2, before starting to plateau from day 2 (Fig. 8*A*). The difference between the mean intensities (HU) within and outside brain tissues increased in both species, from day 0 to 10 (difference ranging from −357.45 to 10,743.46 in *C. punctatum*) and from day 0 to 4 (difference ranging from −1148.02 to 24,303.86 in *C. auratus* (Fig. 8*C*,*D*). In *C. punctatum*, the difference values remained close to zero until day 4, then increased steadily between days 4 and 8, before starting to plateau from day 8 (Fig. 8*C*). These values are differences between mean intensity of brain tissues minus mean intensity of background (inner brain case), across staining times. For the first staining times, the stain penetrated the brain case before reaching the brain tissues, hence creating the negative differences observed at the start (T_0–1_). In *C. auratus*, the difference values were close to zero at T_0_, increased immediately after the specimens were placed into stain compared with *C. punctatum*, then steadily increased between days 1 and 3, before starting to plateau from day 3 (Fig. 8*D*).

#### Measuring staining homogeneity in the whole brain

For the whole brain (*n *=* *4 slices pooled), mean P3 indicated that there was initially no difference in staining of the central nervous tissues for both model species at T_0_, because the samples were unstained. These differences increased markedly as soon as the stain penetrated the edges of the brain in both species, reaching peak differences at day 6 for *C. punctatum* (Fig. 8*E*) and day 2 for *C. auratus* (Fig. 8*F*). The difference values then decreased slightly and plateaued at day 10 for *C. punctatum* and at day 4 for *C. auratus*

### Average linear tissue shrinkage in the brain

**Figure 9.**
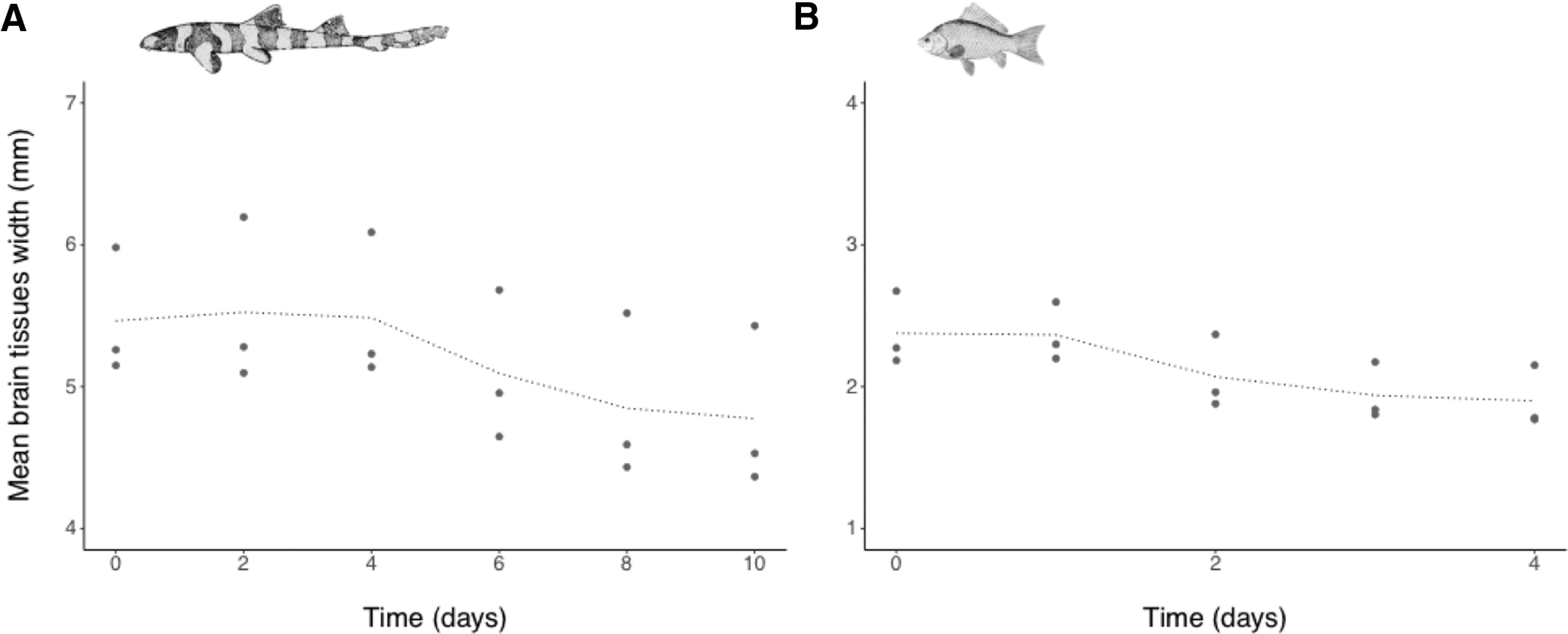
Plots showing the change of width in brain tissues, across all four areas or slices sampled (excluding telencephalon on slice 1 for the shark specimens; *n *=* *4 four slices pooled). ***A***, *C. punctatum* (*n *=* *3). ***B***, *C. auratus* (*n *=* *3). Data points and dotted lines as in Fig. 3.

The mean tissue width for the overall brain in each species (*n *=* *4 slices; *n* = 3 specimens pooled) shows that tissue shrinkage occurred earlier in *C. auratus* than in *C. punctatum*. Tissue shrinkage occurred from day 4 in *C. punctatum* (Fig. 9*A*) and from day 2 in *C. auratus* (Fig. 9*B*), which mirrored the change in contrast levels associated with the onset of stain uptake as shown in Figure 8. Mean tissue width decreased from 5.46 ± 0.45 mm SD (T_0_) to 4.78 ± 0.57 mm SD (T_10_) in *C. punctatum* and from 2.38 ± 0.26 mm SD (T_0_) to 1.90 ± 0.22 mm SD (T_4_) in *C. auratus* (Fig. 9). The shrinkage was more pronounced between days 4 and 8 for *C. punctatum* and days 1 and 2 for *C. auratus*. The width of brain tissues (*n *=* *4 slices pooled in each specimen) appeared to reduce between days 6 and 10 for *C. punctatum*. For *C. auratus*, shrinkage decreased between days 2 and 3 and plateaued at day 4. Because of the difference in the size (width) of the four brain regions sampled on the different slices, width values were sometimes substantially higher or lower than the interquartile range values, thus appearing as outliers following the same trend (Fig. 9).

When considering slices and individuals separately, tissue shrinkage appeared to vary between brain areas (*n *=* *4 slices), individuals (*n *=* *6), and species (*n *=* *2; Table 6). Percentage rates ranged from 10.73 ± 2.87% SD in the cerebellum to 16.79 ± 5.82% SD in the medulla oblongata of *C. punctatum* and from 8.83 ± 2.94% SD in the OBs to 23.53 ± 2.96% SD in the cerebellum of *C. auratus*. The overall, linear tissue shrinkage observed in the brain was estimated to be 13.54 ± 4.75% SD for *C. punctatum* (*n *=* *3) and 17.77 ± 7.25% SD for *C. auratus* (*n *=* *3) (Table 6).

### Staining quality in the peripheral nervous system

**Figure 10.**
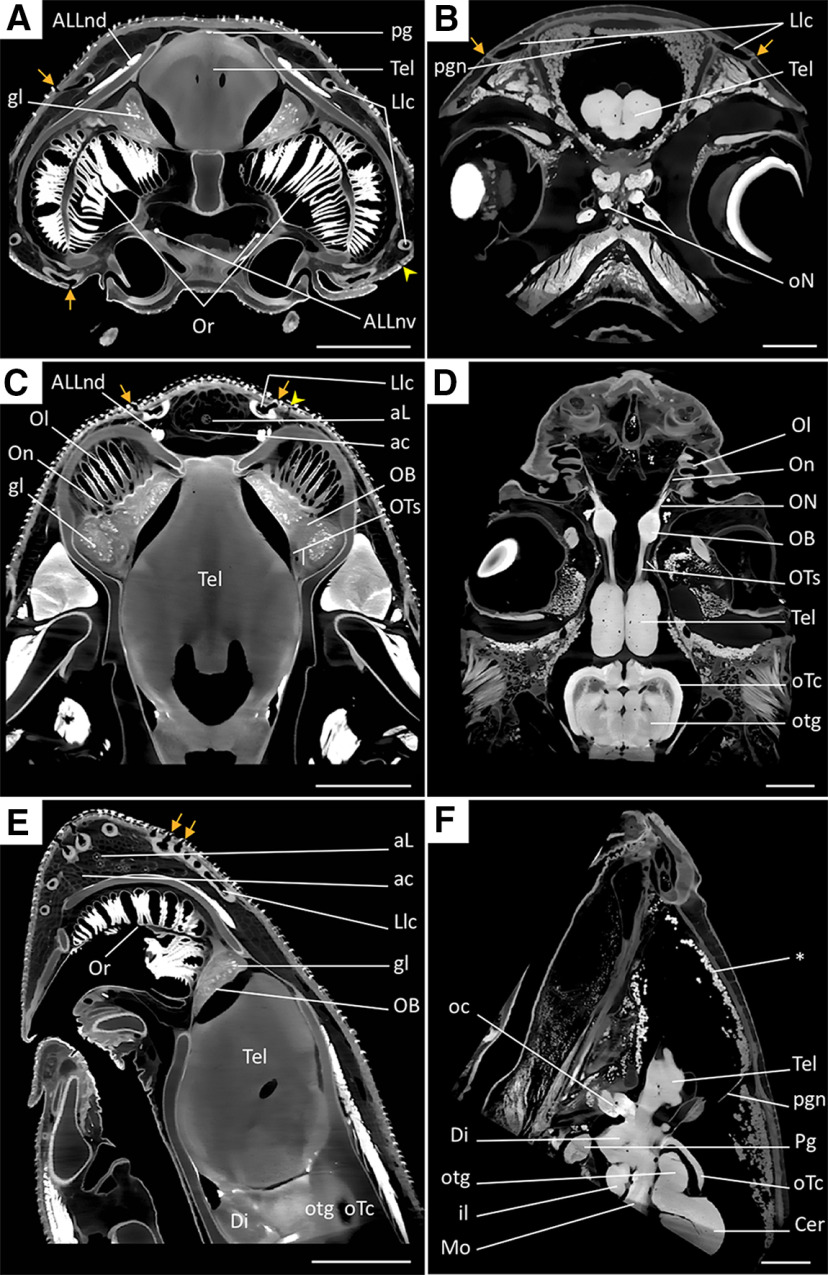
Orthoslices of the filtered volume rendered data from the anterior head of *C. punctatum* (***A***, ***C***, ***E***) and *C. auratus* (***B***, ***D***, ***F***), acquired at higher resolutions (under scanning parameters presented in Table 2), showing frontal (***A***, ***B***), dorso-ventral (***C***, ***D***), and sagittal (***E***, ***F***) slices. The presented orthoslices show the levels of staining in several regions of the anterior head of both species. Intensity range was set at 18,000–40,000 for orthoslices ***A***, ***C***, ***E*** and 10,000–35,000 for orthoslices ***B***, ***D***, ***F***. Scale bars = 5 mm. From top left to bottom right: gl, glomeruli structures in the olfactory bulbs; ALLnd, dorsal root of the anterior lateral line nerve (trigeminal nerve branch V); ALLnv, dorsal root of the anterior lateral line nerve (trigeminal nerve branch VII); Or, olfactory rosette; pg, pineal gland; Tel, telencephalon; Llc, lateral line canals; pgn, pineal gland nerve; oN, optic nerve; On, single olfactory nerve bundle; Ol, olfactory lamella; aL, ampulla of Lorenzini; ac, ampullary canal; OTs, olfactory tracts in the peduncle; ON, olfactory nerve (cranial nerve I); oTc, optic tectum; Di, diencephalon; otg, optical tegmentum; oc, optic chiasma; il, inferior lobe; Mo, anterior extent of the medulla oblongata; *, adipose tissue; Pg, pituitary gland; Cer, cerebellum; orange arrows indicate lateral line canals openings (pores); yellow arrowheads indicate electrosensory (or ampullary) pores.

Effective iodine staining was also observed in the peripheral nervous system, with finer-scale intra-tissue information clearly visible in the higher resolution scans (11–12 μm for *C. punctatum*, 10 μm for *C. auratus*) of the forebrain and surrounding head (Fig. 10).

In *C. punctatum*, the ampullary (electrosensory system) and lateral line (mechanosensory system) pores at the surface of the skin were well-defined, where their relative size (opening diameter) and the length of their canals was also distinguishable (Fig. 10*A*,*C*,*E*). The ampullary sacs (or ampullae of Lorenzini) and their alveoli were also evident, and the number of alveoli easily discernible in the rostral (Fig. 10*C*,*E*) and hyoid (Movie 1, Movie 2) ampullary clusters. The staining even revealed fine structures in the ascending olfactory pathways, such as the number of olfactory lamellae on either side of the middle raphe (approx. 40), the single olfactory nerve bundles projecting from each single fold of the olfactory lamellae into the OBs (Fig. 10*C*), glomerular clusters within the OBs (Fig. 10*A*,*C*,*E*), and the ventricular space within the olfactory peduncles (rhinocele) (Fig. 10*C*). Other afferent nerves were identified, such as the dorsal and ventral roots of the anterior lateral line nerve (ALLnd and ALLnv, trigeminal nerve branch V and VII, respectively) and afferent axons from the rostral ampullary clusters and the lateral line canals, carrying both electrosensory and mechanosensory information, respectively (Fig. 10*A*,*C*,*E*).

Movie 1. Antero-posterior orthoslice video of the anterior head region of *C. punctatum*, edited in Avizo. 10.1523/ENEURO.0076-20.2020.video.1

Movie 2. Dorso-ventral orthoslice video of the anterior head region of *C. punctatum*, edited in Avizo. 10.1523/ENEURO.0076-20.2020.video.2

In *C. auratus*, the lateral line pores and canals were also clearly visible at the skin surface (Fig. 10*B*). The ascending olfactory pathway and its different components were also revealed, including the olfactory rosettes, which were comprised of 10–12 olfactory lamellae, bundles of olfactory axons emanating from each lamella to form the olfactory nerve (cranial nerve I, in contrast to *C. punctatum*), the OBs, two main olfactory tract bundles (lateral and medial), and the telencephalon (Fig. 10*D*). Although the glomerular structures were not visible at high resolution in *C. auratus*, the ventricular space extending from the telencephalon into each olfactory peduncle, and the two main olfactory tracts (medial and lateral), were still distinguishable (Fig. 10*D*). The optic nerves, optic chiasma (Fig. 10*B*,*F*), optic tectum, tegmentum, diencephalon, and pituitary gland were all recognizable (Fig. 10*D*,*F*). At these optimized staining times, the pineal gland and/or its innervation was clearly visible in both species (Fig. 10*A*, for *C. punctatum*, *B*,*F*, for *C. auratus*).

## Discussion

The present study demonstrates a novel application of the diceCT technique for imaging the central and peripheral nervous systems of representative model species from two fish taxa. Specifically, we developed a protocol to determine adequate contrast between the brain and surrounding tissues, facilitating subsequent segmentation of the brain for volumetric analysis. We achieved this by identifying optimal staining times based on an assessment of three quantitative parameters monitored across four brain areas as a proxy for the whole brain. Additionally, we documented the level of linear tissue shrinkage for the different brain areas and the overall brain and reported on the staining quality in peripheral nervous tissues.

### Determination of optimal staining times in the CNS

Optimal staining for X-ray μCT can be defined as the point at which strong intertissue differentiation and intratissue homogeneity is achieved. While most anterior regions of the brain were optimally stained at earlier time points in both species, the most posterior regions required a later time point to achieve the same result. As the aim of this study was to facilitate the semi-automated segmentation of the whole brain from the volume rendered data, later time points were deemed appropriate (10 d for *C. punctatum* and at 4 d for *C. auratus*). Indeed, the results show that optimal staining was reached at earlier time points for anterior brain regions, which thus started to show overstaining for subsequent time points; therefore, the staining and scanning procedure was terminated as soon as the three conditions for staining optimization were met at the posterior part of the brain, as defined in this study (first time point to meet P1–P3 criteria). However, these results depend on the staining parameters in relation to the size of our study specimens. Other studies imaging the head of animals, across diverse taxa, have also used Lugol’s iodine (I_2_KI) with a range of staining parameters. In these 14 studies, stain concentrations varied from 2.25% w/v I_2_KI in the mouse *Mus musculus* (Jeffery et al., 2011) to 40% w/v in the common adder *Vipera berus* (Gignac et al., 2016), and achieved adequate staining within 7 h to 100 d, respectively. Based on the range of concentrations used (Gignac et al., 2016) for a variety of specimens (Amphibia, Reptilia, Aves, Mammalia) and considering the size of our study species and our intent to monitor the stain progression in different areas of the brain, we opted for a 3% Lugol’s solution (1% w/v I_2_, 2% w/v KI). We chose a fixed concentration to be able to monitor the progression of stain uptake over time, as it has not yet been documented in our two study species.

While the present study estimated staining times necessary to visualize the entire brain, it may be advisable to adapt the staining times provided, depending on the regions of interest. For instance, optimal staining of the OBs (where there is a minimal barrier to stain penetration) required only 6 d for *C. punctatum* and 2 d for *C. auratus* (Figs. 2, 4). For the pineal gland in these species, optimal staining times would be expected to be the same as for the whole brain (10 d for *C. punctatum*; 2 d for *C. auratus*), provided the specimens are within the same size range (as seen on slice 1 in Figs. 1, 2 for *C. punctatum* and *C. auratus*, respectively). For the telencephalon, it is important to note that the larger part (posterior extent) sampled on slices 2 in our study was still slightly under-stained at the last time point in *C. punctatum* (Figs. 3, 6); longer immersion times in Lugol’s solution would be required to allow further stain uptake in this brain region. Consideration should also be given to the size range of specimens used for each species, because any individuals that significantly differ in size would require a different staining regime to achieve similar results. The different head and/or body morphology would also play a role in stain uptake, as stain penetration is subject to the volume of tissues to infiltrate. Differences in the thickness of the skin, or the material and arrangement of scales, may also have an influence on the stain penetration rates. Indeed, we observed a slower penetration of stain in *C. punctatum*, which could be the combined result of a larger sized head, but also thicker skin and the presence of mineralized denticles, instead of scales as in *C. auratus*.

Moreover, intraspecific variation can be common in biological samples of similar sizes. Our results showed that such variations were present, mainly in *C. punctatum*, for almost all the parameters measured, and specifically over later staining time points, although individuals were chosen to be within a limited size range. Despite the consistent sampling for the repeated measures collected across time points, there was one individual per species for which the staining was slightly different. This could be due to a difference in age, sex or to slight differences in size within sex, although this was not within the scope of our study, and therefore not tested. Nonetheless, it is important to note that ontogenetic variation in brain size is known to occur in both these fish taxa (Brandstätter and Kotrschal, 1990; Wagner, 2003; Lisney et al., 2007, 2017, 2018), as fish display indeterminate growth (Sebens, 1987; Mommsen, 2001) and adult neurogenesis (Zupanc, 2001; Kizil et al., 2012). Furthermore, some studies have shown sexual dimorphism in the nervous systems of fish, e.g., in their olfactory (Kotrschal et al., 1998; Kasumyan, 2004) and electrosensory (Kempster et al., 2013) systems. However, we are not able to draw any definitive conclusions on sexual dimorphism and ontogenetic variation based on our use of immature individuals and our limited sample size. Although the overall trends were similar, a certain degree of biological variation is to be expected, and will be an important consideration for future studies, especially for those with limited sample size.

This study monitored staining by sampling with a slice-based approach (i.e., four line probes, across four brain areas, with *n *=* *500 sampled pixels per slice). The positions of the slices and line probes were chosen to cover various neural tissues (and avoiding the ventricular spaces), to represent the whole brain. Although our extrapolation from a limited number of pixels may have led to overrepresentation or underrepresentation of the staining outcomes, when considering specific areas of interest, we believe that the use of the same number of replicates in a repeated-measures design across staining time points enabled us to account for potential intraspecific variation, which has not always been incorporated into previous studies assessing stain progression and staining quality in a quantitative manner. Image optimization is particularly important when visualizing new tissues, regions, or species of interest, for which no or little indication of staining times are available. The fact that the stain was replaced after each scanning time point in our scanning regime also ensured the concentration gradient remained consistent between time points. We did not use a 3D approach for our sampling, due to the adjustments needed to collect voxel data of the inner brain (excluding the ventricles) consistently across time points, i.e., during staining with the associated shrinkage. Sampling in 3D would require automated segmentation, taking into consideration the rate of shrinkage between time points (i.e., the amount of shrinkage per day), which is nonlinear over time as shown in our results (see result section: Average linear tissue shrinkage in the brain). Although considered, 3D sampling was outside the remit of this study.

### Levels of tissue shrinkage in the CNS

We found that the percentage of linear tissue shrinkage was not constant through time but followed a similar trend in both species. The greatest degree of shrinkage observed in the brain of both species was concurrent with the onset of stain uptake. Shrinkage was highest when the solution had started to penetrate brain tissue, which became more X-ray opaque, after 2–4 d, then decreased to eventually plateau at later time points, from day 3 to 4, as found by Vickerton et al. (2013). As for the staining parameters, shrinkage also occurred earlier in *C. auratus*. Previous studies have indicated that the degree of tissue shrinkage due to the staining process, as opposed to the shrinkage produced by sample preservation, was directly influenced by the concentration of I_2_KI used in solution, showing that higher levels of shrinkage occurred, and more rapidly, at higher concentrations (Tahara and Larsson, 2013; Vickerton et al., 2013; Gignac and Kley, 2014). In the present study, the different levels of shrinkage observed are thought to be minimized because we chose to use an aqueous-based solution of I_2_KI (Lugol’s iodine), on samples fixed and stored in an aqueous solution of glutaraldehyde, as opposed to alcohol-based solutions (I_2_E or I_2_M; Buytaert et al., 2014). We did not test, optimize, nor perform comparisons of levels of shrinkage in different brain regions with these other solutions (or using different buffers), but this could be the subject of future research.

We reported a mean overall shrinkage of ∼14% in *C. punctatum* and ∼18% in *C. auratus* (i.e., across the four brain areas sampled, between T_0_ and end time point). As the same staining solution was applied, under the same conditions, for both species, the difference in mean shrinkage at the level of the whole brain was likely linked to differences in the morphology, structure (skin), and size of the head, as previously mentioned, as well as the specimen and brain sizes. For example, we noticed that central nervous tissues generally appeared more stained in *C. auratus* specimens, when displaying comparable intensity ranges (Fig. 8). The higher level of staining seen in *C. auratus* would have likely been due to a faster stain penetration and, therefore, could have resulted in the greater overall shrinkage observed. Whether the stain penetration is faster in the goldfish because of a smaller sample size or a difference in osmolality compared with the shark, which may be more isotonic to the Lugol’s solution, needs further research. Nonetheless, the solution was hypertonic to both (net water movement out of the fish) leading to shrinkage. The degrees of shrinkage found are considerably lower than the average shrinkage reported by Buytaert et al. (2014; 39% in fresh, unfixed specimens of juvenile rabbits *Oryctolagus cuniculus*) and Hedrick et al. (2018; 38% in museum-preserved bat specimens from five noctilionoid species), both of which used similar concentrations of I_2_KI (3% and 2.5%, respectively). However, Hedrick et al. (2018) found a much lower degree of shrinkage (6%) in field-collected, freshly-fixed bat specimens, although they used brain/endocast ratios to determine the initial brain tissue volume and assessed subsequent shrinkage. Our study focused on assessing the shrinkage due to the staining procedure, not the combination of preservation and staining, or from fresh volumes. We could have considered assessing the initial, unfixed brain volume using MRI, had we not used specimens already preserved, which may have resulted in higher levels of shrinkage. Buytaert et al. (2014) used samples of undifferentiated brain tissue (i.e., no specified brain area) in mammals. While an endocast can be used as a proxy for brain volume in mammals, this is not as reliable in fish, as the brain case is not occupied entirely by the brain in some species (Kruska, 1988; Yopak and Frank, 2009); for instance, adipose tissue is prominent in the brain case of *C. auratus* (Fig. 10). Overall, these considerations limit the comparisons that can be made.

Interestingly, there were substantial intraspecific and interspecific variations in the percentage of shrinkage between brain areas, which is likely due to differences in nervous tissue type and composition. Indeed, varying cell densities, size, or types (neuronal vs non-neuronal cells or myelinated vs unmyelinated axons) may have locally influenced the diffusion rate or tonicity properties of the Lugol’s solution (I_2_KI in distilled water; Weisbecker, 2012; Vickerton et al., 2013; Gignac and Kley, 2018). In solution, I_2_ and KI tend to form iodine trimers or triiodides (I_3_^–^) and potassium ions (K^+^), which form I_3_^–^-K^+^ bonds (Degenhardt et al., 2010), as well as more complex polyiodide products, such as I_5_^–^, I_9_^–^, and maybe longer chains (Yu et al., 1996; Li et al., 2016). Elementary iodide (I_i_^–^) and potassium triiodide (I_3_K) products bind preferentially to carbohydrates, such as glycogen (Bock and Shear, 1972; Metscher, 2009b), and lipids (Babaei et al., 2016), which are naturally present in varying amounts within different types of soft tissues, and especially nervous tissues (Gignac and Kley, 2014). Li et al. (2016) demonstrated the positive correlation between glycogen concentration and grayscale values experimentally, within several tissue types, including nervous tissue. Although their study compared two iodine-based solutions, including I_2_KI in 10% neutral buffered formalin, their results confirmed that glycogen is one of the major absorbents of iodine that enhance contrast during staining (Lecker et al., 1997; Jeffery et al., 2011; Li et al., 2015). Additionally, as the membrane of Schwann cells ensheathing myelinated axons is characterized by high proportions of lipids compared with white matter (Morell and Quarles, 1999), brain regions with higher densities of myelinated axons (e.g., the glomerular layer in the OBs) would likely yield a higher level of shrinkage, due to the high osmolarity of the staining solution. This may explain why, for instance, we observed a greater shrinkage in the OBs of *C. punctatum* compared with *C. auratus*. As samples remain osmotically active after fixation, the fact that we used species from relatively different osmotic environments (one saltwater and one freshwater), may have an impact on shrinkage. This hypothesis could also be tested in future studies. Together, the binding affinities of iodine and differential nervous tissue composition would produce differential shrinkage levels across the brain.

### Applications and recommendations for future research

This study highlights the potential applications of contrast-enhanced X-ray imaging in comparative neuroanatomy. We demonstrate that using Lugol’s solution, as per the diceCT method, is effective at enhancing contrast to visualize not only the brain but also afferent nerves and most peripheral organs. The effectiveness of iodine-based stains mainly relies on the differential solubility and affinity of iodide products in solution. This explains why Lugol’s solution also enables visual differentiation of myelinated and unmyelinated neuronal tissues within both the peripheral and central nervous systems *in situ* (Gignac and Kley, 2014, 2018), which makes the diceCT technique a valuable tool in the field of comparative neuroanatomy, particularly volumetric analysis of the brain. Recently, neuroecological studies have used MRI to assess the volume of the brain and its major subdivisions in cartilaginous (Yopak and Frank, 2009; Yopak et al., 2019) and bony fish (Ullmann et al., 2010a,b). In comparison, diceCT imaging offers a relatively rapid and cost-effective alternative to MRI, to achieve similar resolutions. Currently, μCT has the capacity to achieve higher resolutions compared with MRI (Gignac and Kley, 2018), thereby providing more detail of the fine structure of the neuroanatomy of sensory systems.

However, the use of iodine-based solutions, such as aqueous I_2_KI, causes a notable level of tissue shrinkage (i.e., >10%), even at low concentrations (≤3%, as in this study; Degenhardt et al., 2010; Vickerton et al., 2013; Buytaert et al., 2014; Li et al., 2015; Bribiesca-Contreras and Sellers, 2017), which is likely greater than with the contrast agents used for MRI; although this remains to be tested. Also, the shrinkage observed in this study may be reduced if a lower (w/v) concentration of iodine was used, but this would likely increase the staining times. Specimens stained with iodine-based agents also remain chemically altered, despite de-staining techniques being available, thereby limiting the possibility of further histologic investigations (Gignac et al., 2016; Gignac and Kley, 2018). Future research should seek to compare the staining performance, level of resolution and degree of tissue shrinkage in the brain of fish specimens from different taxa, using both imaging techniques. Especially in the case of a limited sample size, or for research focusing on volumetric analyses, preliminary assessment of the levels of shrinkage for specific tissues or brain regions of interest is essential, and the calculation of tissue-specific correction factors, as suggested by Vickerton et al. (2013), would be required. The approach offered in this study, where the outputs of least-squared multiple regressions for three main variables (initial sample size, I_2_KI concentration and incubation time) were used to correct for tissue shrinkage, is the only option available to date for assessing shrinkage conditions in specific tissues, mathematically. However, the formula for correction of tissue shrinkage given by the above study requires knowledge of the initial brain volume in the same specimens, which was not possible to measure without staining in our study design, and was thus out of the scope of this study. Shrinkage has been largely overlooked in volumetric studies, with only a limited number of studies that have provided an indication of the level of overall brain volume shrinkage due to staining (Buytaert et al., 2011, 2013; Rau et al., 2013).

We hope this study serves as a useful guide for the application of diceCT in future research on the sensory neuroanatomy of fish. These methods may enable volumetric analyses of different brain areas, which will help to predict the relative importance of different sensory systems across species (Lisney et al., 2007; Kotrschal et al., 2017) and/or other quantitative morphometric analyses of a diversity of tissue types (Weinhardt et al., 2018). Further, diceCT imaging may offer a more accurate approach for *in situ* measurements rather than volumetric approximation methods *ex situ*, such as previous weighing, histologic, or fitted ellipsoid approaches (Lisney et al., 2007; Ullmann et al., 2010b). diceCT could also enable studies of the organization of hard tissue structures, including the skin denticles of cartilaginous fish, the jaw musculature or cartilage, cranial morphology, and tooth mineralization (Jambura et al., 2019). Museum specimens could be also scanned to create web-accessible virtual collections or anatomic atlases, such as online repositories of digital material from both MRI (Ullmann et al., 2010c) and CT data (Yopak et al., 2018), for research and/or educational purposes or to advance taxonomic, evolutionary studies, i.e., The Chondrichthyan Tree of Life Project (CToL; https://sharksrays.org) and vertebrate paleontology records (Benton, 2014; Lessner and Stocker, 2017).

Overall, diceCT offers a versatile approach for the visualization of a range of tissue types, including the brain. Our study represents one of only a few to study the brain in fish taxa. The other two fish species examined with diceCT are the northern clingfish *Gobiesox maeandricus* (Kleinteich et al., 2014) and the elephantnose fish *Gnathonemus petersii* (Gignac and Kley, 2018). While the former study used this imaging technique to assess the mechanical aspects and functional role of the suction cup in *G. maeandricus*, the latter highlighted the qualitative value of diceCT for comparative neuroanatomy. Here, establishing optimal staining times was necessary before conducting subsequent volumetric analyses on the two fish models. To date, no study has used a quantitative approach to assess stain uptake when using diceCT with Lugol’s solution in any species of fish. Gignac and Kley (2014) quantified staining intensities in a range of cephalic tissues including the brain, in the American alligator *Alligator mississippiensis* and the emu *Dromaius novaehollandiae*, with Lugol’s solution. Li et al. (2016) compared the staining performance of two commonly used approaches for diceCT (I_2_Kl-formaldehyde and I_2_E) in the cephalic region of the Chilean tinamou, *Nothoprocta perdicaria*. However, they did not use Lugol’s solution (aqueous I_2_Kl). Unlike previous studies, our research is the first to use the same number of individuals across time points consistently, and use repeated measures to optimize contrast in the brain of fish species from two taxa; a marine fish species, specifically a chondrichthyan, compared with a freshwater teleost. This study represents the first comprehensive use of diceCT to investigate detailed neuroanatomy of two fish model species.

## Synthesis

Reviewing Editor: Christine Portfors, Washington State University Decisions are customarily a result of the Reviewing Editor and the peer reviewers coming together and discussing their recommendations until a consensus is reached. When revisions are invited, a fact-based synthesis statement explaining their decision and outlining what is needed to prepare a revision will be listed below. The following reviewer(s) agreed to reveal their identity: Jaimi Gray.

The manuscript is well-written, rigorous in its approach and provides a useful tool for all fish species. Addressing the reviewers’ concerns would increase the impact of the manuscript. In particular, addressing the sex of the goldfish, how other researchers might optimize this technique for other species, a comparison of their technique with other iodine stains and some modifications to the figures would improve this manuscript.

Reviewer 1:

The manuscript titled “diceCT: a valuable technique to study the nervous system of fishes” describes a detailed protocol for micro-computerized tomography (μCT) using an iodine based contrast agent, Iodine Potassium Iodine (I2KI, a.k.a. Lugol’s Solution). The authors demonstrate the effectiveness of this approach using two distinct fish species, the bony fish, C. auratus (the common goldfish) as well as the cartilaginous fish, C. punctatum (the brownbanded bamboo shark). The authors demonstrate that Lugol’s solution is effective in staining fish species, and, for the species studies, they examine optimal staining time across different tissue regions, evaluate the degree of shrinkage caused by the iodine-based stain, and examine the effectiveness of staining in peripheral tissue. Additionally, the authors provide code (written in R; both code and R or R-Studio are freely available) for the rigorous analysis of diceCT images.

High resolution brain atlases have been commonplace for model systems such as rat and mouse, and more recently, their application has been extended to other models such as Drosophila and zebrafish. However, for fish species, whole-brain atlases are typically developed for larval forms, as the large size and opacity make imaging the entire brain difficult. Despite this, brain atlases are becoming increasingly important as catalogues of neuronal anatomy, neuronal activity and genetic identity of specific neurons. MRI is a valuable approach, but difficult and expensive to conduct, and CT provides a cost-effective measure, but resolution is not as good.

The current manuscript provides a useful tool for all fish species. Moreover, the manuscript is well-written and easy to understand, and rigorous in its approach. The limitations to the study as it stands, and that should be addressed before acceptance, are:

1) The authors do an exceptional job at detailing optimal conditions for contrast-based imaging using Lugol’s solution, yet it is unclear whether Lugol’s is superior to other iodine-based stains. The authors imply that Lugol’s will cause less shrinkage, yet this is not studied. I would like to see a comparison of other Iodine stains to demonstrate that Lugol’s is indeed better. Alternatively, the authors could describe in detail the evidence demonstrating that Lugol’s is indeed a better stain that causes less shrinkage.

2) Similarly, the value of this manuscript is in the wide application of brain imaging in fish species, yet only two species of fish are examined. While I would have liked to have seen a comparison with standard model systems such as zebrafish, it is unreasonable to ask at this point; however, the authors could describe how researchers can use this to better optimize staining for their own species. For example, is there a correlation between staining times and other parameter (size of brain, boney vs. cartilaginous, etc.) that could be used to predict better staining conditions?

3) Regarding the staining times, the optimal staining point was (for some z-sections) unambiguously chosen as the last time point, thus it is unclear whether staining would have improved or not had the staining been left for longer. I would assume there is either saturation or an optimal time point, but if they did not go beyond those stages, then how do you know you have hit a plateau? some of the graphs are still in a positive rising phase. Could the authors either show that staining does saturate (via an asymptote on the curve) or explain why this was stopped?

4) Why was sex not determined for Goldfish? It seems straight-forward to sex goldfish, and this would add a lot to the study, esp. since the authors claim that there could be sex-dependent differences in staining. The authors should either evaluate how sex impacts staining in goldfish, or give rationale for ignoring sex.

5) Similarly, it is unfair to state that there could be sex-dependent differences in staining when only one male was examined the bamboo shark. This, along with point 4 could be addressed simultaneously by addition of goldfish sex data, or by including additional males to the bamboo shark study, or both.

Reviewer 2:

I particularly appreciate that the authors address the issue of shrinkage and provide a measure of how much has occurred during their staining prototcol. They address this well in the discussion, but it could perhaps be improved by mentioning that the shrinkage observed in this study could perhaps be reduced if a lower w/v concentration of iodine was used here.

The figures in this paper are beautiful! But for figures 5a and 5b, I found it hard to distinguish between the lines on the plots, perhaps different coloured lines, or different line styles might make this more clear.
