# Supplementary material for: diceCT: A Valuable Technique to Study the Nervous System of Fish
Source: eNeuro. 2020 Aug 13;7(4):ENEURO.0076-20.2020. doi: 10.1523/ENEURO.0076-20.2020 (PMC7642124; doi:10.1523/ENEURO.0076-20.2020)
Supplement: Extended Data 1 — R code used for the peak analysis, to extract the data required for analysis. Download Extended Data 1, ZIP file. [file enu-eN-MNT-0076-20-s01.zip › CT-fish-brain-master/R Code for peak analysis.docx]

library("pracma")

####### CP #######

# Define some constants

specimen <- 'CP6'

# The specimen you wish to process

filepath <- '/Users/Desk/'

# Directory containing the csv files

number.edges.to.find <- c(8,4,4,8)

# Defines the number of edges to find in lineprobe (aka slice) 1, 2, 3 and 4

first.timepoint <- 2;

# Column containing the first timepoint of interest

last.timepoint <- 7;

# Column containing the last timepoint of interest

min.peak.distance <- 5;

# Read the "findpeaks" help

# Data frame to hold the data for the spreadsheet of measurements

result<-data.frame(slice=integer(),

time=integer(),

edge1=double(),edge2=double(),edge3=double(),edge4=double(),edge5=double(),edge6=double(),edge7=double(),edge8=double(),

grad1=double(),grad2=double(),grad3=double(),grad4=double(),grad5=double(),grad6=double(),grad7=double(),grad8=double(),

median1=double(),median2=double(),median3=double(),median4=double(),median5=double(),median6=double(),median7=double(),

iqr1=double(),iqr2=double(),iqr3=double(),iqr4=double(),iqr5=double(),iqr6=double(),iqr7=double(),

first.quartile1=double(),first.quartile2=double(),first.quartile3=double(),first.quartile4=double(),first.quartile5=double(),first.quartile6=double(),first.quartile7=double(),

third.quartile1=double(),third.quartile2=double(),third.quartile3=double(),third.quartile4=double(),third.quartile5=double(),third.quartile6=double(),third.quartile7=double(),

mean1=double(),mean2=double(),mean3=double(),mean4=double(),mean5=double(),mean6=double(),mean7=double(),

sd1=double(),sd2=double(),sd3=double(),sd4=double(),sd5=double(),sd6=double(),sd7=double(),

width1=double(),width2=double(),width3=double(),width4=double(),width5=double(),width6=double(),width7=double(),

middle_intensity1=double(),middle_intensity2=double(),middle_intensity3=double(),middle_intensity4=double(),middle_intensity5=double(),middle_intensity6=double(),middle_intensity7=double())

# Begin processing data for the chosen specimen

for (lineprobe.number in 1:4) {

filepathname <- paste(filepath,specimen,'_slice',lineprobe.number,'_T0-240.csv',sep="") # Construct the complete file path and name

# Read in the data for the this lineprobe (excluding the header)

# - First column contains the cumulative length of the probe line in microns

# - Remaining columns are intensity profiles for each timepoint: no stain T0, T1, T2...

print(filepathname)

lineprobe.data <- read.csv(filepathname,header=TRUE)

# Convert the distance values along the lineprobe to mm

distance <- lineprobe.data[,1]/1000

# Plot the intensity profile for this lineprobe for all timepoints

quartz()

matplot(distance,lineprobe.data[,-1], type="l", xlab='mm',ylab='Intensity')

nn <- length(lineprobe.data[,-1])

legend("topright", colnames(lineprobe.data[,-1]),col=seq_len(nn),cex=0.8,fill=seq_len(nn))

# Process each timepoint separately

for (timepoint in first.timepoint:last.timepoint) {

single.profile <- lineprobe.data[, timepoint]

# Smooth the profile

smoothed.single.profile <- smooth.spline(single.profile, spar = 0.2)$y

# Compute the magnitude of gradient for the smoothed profile

profile.grad <- conv(smoothed.single.profile, c(-2, 0, 2)) # Convolve with a central differences mask

magnitude.of.gradient <- abs(profile.grad[c(-1, -501)]) # Crop

magnitude.of.gradient[c(1, length(magnitude.of.gradient))] <- 0 # Set both ends to zero

# Locate the peaks in the magnitude of gradient to the LHS and to the RHS of the midpoint

length.of.probe <- length(distance)

midpoint.of.probe <- floor(length.of.probe)/2

all.LHS.peaks <- findpeaks(magnitude.of.gradient[1:midpoint.of.probe], minpeakdistance = min.peak.distance, sortstr = TRUE)

all.RHS.peaks <- findpeaks(magnitude.of.gradient[midpoint.of.probe+1:length.of.probe], minpeakdistance = min.peak.distance, sortstr = TRUE)

all.location <- c(all.LHS.peaks[, 2],all.RHS.peaks[,2]+midpoint.of.probe) # concatenate the LHS and RHS locations

all.peak.height <- c(all.LHS.peaks[, 1],all.RHS.peaks[, 1]) # concatenate the LHS and RHS heights

selected.LHS.peaks <- matrix(all.LHS.peaks[1:(number.edges.to.find[lineprobe.number]/2),],ncol=4) # keep only the required number of "biggest" peaks on the LHS

selected.RHS.peaks <- matrix(all.RHS.peaks[1:(number.edges.to.find[lineprobe.number]/2),],ncol=4) # keep only the required number of "biggest" peaks on the RHS

selected.LHS.peaks <- matrix(selected.LHS.peaks[order(selected.LHS.peaks[, 2]), ],ncol=4) # Order peaks by peak location

selected.RHS.peaks <- matrix(selected.RHS.peaks[order(selected.RHS.peaks[, 2]), ],ncol=4) # Order peaks by peak location

location <- c(selected.LHS.peaks[, 2],selected.RHS.peaks[,2]+midpoint.of.probe) # concatenate the LHS and RHS locations

peak.height <- c(selected.LHS.peaks[, 1],selected.RHS.peaks[, 1]) # concatenate the LHS and RHS heights

answer <- "n"

while (substr(answer, 1, 1) == "n") {

# Plot the profile and the result after smoothing

quartz()

plot(distance, single.profile, type = "l", xlab = "mm", ylab = "Intensity", ylim = c(0, max(single.profile)), lab = c(25, 5, 7))

lines(distance, smoothed.single.profile, col = "green")

# Overlay the plot of the magnitude of gradient

lines(distance, magnitude.of.gradient, col = "red")

# Plot all of the identified peaks

lines(distance[all.location], all.peak.height, type = "p")

# Highight the selected peaks

for (j in 1:length(location)){

x<-c(distance[location[j]],distance[location[j]])

y<-c(0,max(smoothed.single.profile))

lines(x,y,type="l") # plot a vertical line for each peak

}

cat("Are you satisfied with the selected peaks? ")

answer<-readLines(con=stdin(),n=1)

if (substr(answer, 1, 1) == "n"){

cat("Select the plot window and then select the correct peaks\n")

which<-identify(distance[all.location], all.peak.height, n=number.edges.to.find[lineprobe.number]) # get the user to select the relevant peaks

location <- all.location[which] # keep only the selected locations and peak heights

peak.height <- all.peak.height[which]

peak.height <- peak.height[order(location)] # order by location

location <- location[order(location)]

dev.off() # close the existing plot

}

}

# Compute measurements based on the selected peaks

width<-as.double(rep(NA,max(number.edges.to.find)-1))

median.intensity<-width

mean.intensity<-width

sd.intensity<-width

middle.intensity<-width

iqr.intensity<-width

first.quartile<-width

third.quartile<-width

for (j in 1:(number.edges.to.find[lineprobe.number] - 1)) {

left.edge.index <- location[j]

right.edge.index <- location[j + 1]

middle.index <- floor((right.edge.index - left.edge.index)/2)

width[j] <- distance[right.edge.index] - distance[left.edge.index]

mean.intensity[j] <- mean(smoothed.single.profile[seq(location[j], location[j + 1])])

median.intensity[j] <- median(smoothed.single.profile[seq(location[j], location[j + 1])])

sd.intensity[j] <- sd(smoothed.single.profile[seq(location[j], location[j + 1])])

iqr.intensity[j] <- IQR(smoothed.single.profile[seq(location[j], location[j + 1])])

middle.intensity[j] <- smoothed.single.profile[middle.index]

first.quartile[j]<-quantile(smoothed.single.profile[seq(location[j], location[j + 1])], 0.25)

third.quartile[j]<-quantile(smoothed.single.profile[seq(location[j], location[j + 1])], 0.75)

}

padding <- max(number.edges.to.find)-length(location)

result[nrow(result) + 1,] <- c(lineprobe.number,timepoint,

distance[location],rep(NA,padding),

peak.height,rep(NA,padding),

median.intensity,

iqr.intensity,

first.quartile,

third.quartile,

mean.intensity,sd.intensity,width,

middle.intensity)

lines(distance[floor(diff(location)/2 + location[1:number.edges.to.find[lineprobe.number] - 1])], median.intensity[1:j], type = "p")

}

}

write.csv(result,paste(filepath,specimen,'_T0-240_results_Final.csv',sep=""))

####### GF #######

# Define some constants

specimen <- 'GF22'

# The specimen you wish to process

filepath <- '/Users/Desk/'

# Directory containing the csv files

number.edges.to.find <- c(6,4,4,8)

# Defines the number of edges to find in lineprobe (aka slice) 1, 2, 3 and 4

first.timepoint <- 2;

# Column containing the first timepoint of interest

last.timepoint <- 6;

# Column containing the last timepoint of interest

min.peak.distance <- 5;

# Read the "findpeaks" help

# Data frame to hold the data for the spreadsheet of measurements

result<-data.frame(slice=integer(),

time=integer(),

edge1=double(),edge2=double(),edge3=double(),edge4=double(),edge5=double(),edge6=double(),edge7=double(),edge8=double(),

grad1=double(),grad2=double(),grad3=double(),grad4=double(),grad5=double(),grad6=double(),grad7=double(),grad8=double(),

median1=double(),median2=double(),median3=double(),median4=double(),median5=double(),median6=double(),median7=double(),

iqr1=double(),iqr2=double(),iqr3=double(),iqr4=double(),iqr5=double(),iqr6=double(),iqr7=double(),

first.quartile1=double(),first.quartile2=double(),first.quartile3=double(),first.quartile4=double(),first.quartile5=double(),first.quartile6=double(),first.quartile7=double(),

third.quartile1=double(),third.quartile2=double(),third.quartile3=double(),third.quartile4=double(),third.quartile5=double(),third.quartile6=double(),third.quartile7=double(),

mean1=double(),mean2=double(),mean3=double(),mean4=double(),mean5=double(),mean6=double(),mean7=double(),

sd1=double(),sd2=double(),sd3=double(),sd4=double(),sd5=double(),sd6=double(),sd7=double(),

width1=double(),width2=double(),width3=double(),width4=double(),width5=double(),width6=double(),width7=double(),

middle_intensity1=double(),middle_intensity2=double(),middle_intensity3=double(),middle_intensity4=double(),middle_intensity5=double(),middle_intensity6=double(),middle_intensity7=double())

# Begin processing data for the chosen specimen

for (lineprobe.number in 1:4) {

filepathname <- paste(filepath,specimen,'_slice',lineprobe.number,'_T0-96.csv',sep="") # Construct the complete file path and name

# Read in the data for the this lineprobe (excluding the header)

# - First column contains the cumulative length of the probe line in microns

# - Remaining columns are intensity profiles for each timepoint: no stain T0, T1, T2...

print(filepathname)

lineprobe.data <- read.csv(filepathname,header=TRUE)

# Convert the distance values along the lineprobe to mm

distance <- lineprobe.data[,1]/1000

# Plot the intensity profile for this lineprobe for all timepoints

quartz()

matplot(distance,lineprobe.data[,-1], type="l", xlab='mm',ylab='Intensity')

nn <- length(lineprobe.data[,-1])

legend("topright", colnames(lineprobe.data[,-1]),col=seq_len(nn),cex=0.8,fill=seq_len(nn))

# Process each timepoint separately

for (timepoint in first.timepoint:last.timepoint) {

single.profile <- lineprobe.data[, timepoint]

# Smooth the profile

smoothed.single.profile <- smooth.spline(single.profile, spar = 0.2)$y

# Compute the magnitude of gradient for the smoothed profile

profile.grad <- conv(smoothed.single.profile, c(-2, 0, 2)) # Convolve with a central differences mask

magnitude.of.gradient <- abs(profile.grad[c(-1, -501)]) # Crop

magnitude.of.gradient[c(1, length(magnitude.of.gradient))] <- 0 # Set both ends to zero

# Locate the peaks in the magnitude of gradient to the LHS and to the RHS of the midpoint

length.of.probe <- length(distance)

midpoint.of.probe <- floor(length.of.probe)/2

all.LHS.peaks <- findpeaks(magnitude.of.gradient[1:midpoint.of.probe], minpeakdistance = min.peak.distance, sortstr = TRUE)

all.RHS.peaks <- findpeaks(magnitude.of.gradient[midpoint.of.probe+1:length.of.probe], minpeakdistance = min.peak.distance, sortstr = TRUE)

all.location <- c(all.LHS.peaks[, 2],all.RHS.peaks[,2]+midpoint.of.probe) # concatenate the LHS and RHS locations

all.peak.height <- c(all.LHS.peaks[, 1],all.RHS.peaks[, 1]) # concatenate the LHS and RHS heights

selected.LHS.peaks <- matrix(all.LHS.peaks[1:(number.edges.to.find[lineprobe.number]/2),],ncol=4) # keep only the required number of "biggest" peaks on the LHS

selected.RHS.peaks <- matrix(all.RHS.peaks[1:(number.edges.to.find[lineprobe.number]/2),],ncol=4) # keep only the required number of "biggest" peaks on the RHS

selected.LHS.peaks <- matrix(selected.LHS.peaks[order(selected.LHS.peaks[, 2]), ],ncol=4) # Order peaks by peak location

selected.RHS.peaks <- matrix(selected.RHS.peaks[order(selected.RHS.peaks[, 2]), ],ncol=4) # Order peaks by peak location

location <- c(selected.LHS.peaks[, 2],selected.RHS.peaks[,2]+midpoint.of.probe) # concatenate the LHS and RHS locations

peak.height <- c(selected.LHS.peaks[, 1],selected.RHS.peaks[, 1]) # concatenate the LHS and RHS heights

answer <- "n"

while (substr(answer, 1, 1) == "n") {

# Plot the profile and the result after smoothing

quartz()

plot(distance, single.profile, type = "l", xlab = "mm", ylab = "Intensity", ylim = c(0, max(single.profile)), lab = c(25, 5, 7))

lines(distance, smoothed.single.profile, col = "green")

# Overlay the plot of the magnitude of gradient

lines(distance, magnitude.of.gradient, col = "red")

# Plot all of the identified peaks

lines(distance[all.location], all.peak.height, type = "p")

# Highight the selected peaks

for (j in 1:length(location)){

x<-c(distance[location[j]],distance[location[j]])

y<-c(0,max(smoothed.single.profile))

lines(x,y,type="l") # plot a vertical line for each peak

}

cat("Are you satisfied with the selected peaks? ")

answer<-readLines(con=stdin(),n=1)

if (substr(answer, 1, 1) == "n"){

cat("Select the plot window and then select the correct peaks\n")

which<-identify(distance[all.location], all.peak.height, n=number.edges.to.find[lineprobe.number]) # get the user to select the relevant peaks

location <- all.location[which] # keep only the selected locations and peak heights

peak.height <- all.peak.height[which]

peak.height <- peak.height[order(location)] # order by location

location <- location[order(location)]

dev.off() # close the existing plot

}

}

# Compute measurements based on the selected peaks

width<-as.double(rep(NA,max(number.edges.to.find)-1))

median.intensity<-width

mean.intensity<-width

sd.intensity<-width

middle.intensity<-width

iqr.intensity<-width

first.quartile<-width

third.quartile<-width

for (j in 1:(number.edges.to.find[lineprobe.number] - 1)) {

left.edge.index <- location[j]

right.edge.index <- location[j + 1]

middle.index <- floor((right.edge.index - left.edge.index)/2)

width[j] <- distance[right.edge.index] - distance[left.edge.index]

mean.intensity[j] <- mean(smoothed.single.profile[seq(location[j], location[j + 1])])

median.intensity[j] <- median(smoothed.single.profile[seq(location[j], location[j + 1])])

sd.intensity[j] <- sd(smoothed.single.profile[seq(location[j], location[j + 1])])

iqr.intensity[j] <- IQR(smoothed.single.profile[seq(location[j], location[j + 1])])

middle.intensity[j] <- smoothed.single.profile[middle.index]

first.quartile[j]<-quantile(smoothed.single.profile[seq(location[j], location[j + 1])], 0.25)

third.quartile[j]<-quantile(smoothed.single.profile[seq(location[j], location[j + 1])], 0.75)

}

padding <- max(number.edges.to.find)-length(location)

result[nrow(result) + 1,] <- c(lineprobe.number,timepoint,

distance[location],rep(NA,padding),

peak.height,rep(NA,padding),

median.intensity,

iqr.intensity,

first.quartile,

third.quartile,

mean.intensity,sd.intensity,width,

middle.intensity)

lines(distance[floor(diff(location)/2 + location[1:number.edges.to.find[lineprobe.number] - 1])], median.intensity[1:j], type = "p")

}

}

write.csv(result,paste(filepath,specimen,'_T0-96_results_Final.csv',sep=""))
